# Angiotensin 1–7 modulates molecular and cellular processes central to the pathogenesis of prostate cancer

**DOI:** 10.1038/s41598-018-34049-8

**Published:** 2018-10-25

**Authors:** Kamila Domińska, Piotr Okła, Karolina Kowalska, Dominika Ewa Habrowska-Górczyńska, Kinga Anna Urbanek, Tomasz Ochędalski, Agnieszka Wanda Piastowska-Ciesielska

**Affiliations:** 1 Department of Comparative Endocrinology, Medical University of Lodz, Lodz, 90-752 Poland; 2 Laboratory of Cell Cultures and Genomic Analysis, Medical University of Lodz, Lodz, 90-752 Poland

## Abstract

Angiotensin 1–7 (Ang1–7) is an endogenous bioactive component of the renin-angiotensin system (RAS). In addition to its cardiovascular properties, its anti-proliferative and anti-angiogenic traits are believed to play important roles in carcinogenesis. The present study examines the influence of Ang1–7 on processes associated with development and progression of prostate cancer cells. Our findings indicate that while Ang1–7 (1 nM; 48 h) can effectively reduce cell proliferation in DU-145, it can induce a significant decrease in the expression of *MKI67* in LNCaP. In both cell lines we also observed a reduction in colony size in soft agar assay. A various changes in gene expression were noted after exposure to Ang1–7: those of anti- and pro-apoptotic agents and the NF-kB family of transcription factors, as well as mesenchymal cell markers and vascular endothelial growth factor A (*VEGFA*). In addition, Ang1–7 was found to modulate cell adhesion and matrix metallopeptidase (MMP) activity. Changes were also observed in the levels of angiotensin receptors and sex steroid hormone receptors. Ang1–7 reduced the levels of estrogen receptor alpha gene (*ESR1*) and increased the expression of estrogen receptor beta gene (*ESR2*) in all prostate cancer cells; it also up-regulated androgen receptor (*AR*) expression in androgen-sensitive cells but contradictory effect was observed in androgen- irresponsive cell lines. In summary, the results confirm the existence of complex network between the various elements of the local RAS and the molecular and cellular mechanisms of prostate cancerogenesis. The response of cancer cells to Ang1–7 appears to vary dependently on the dose and time of incubation as well as the aggressiveness and the hormonal status of cells.

## Introduction

Angiotensin 1–7 (Ang1–7) is an endogenous, bioactive component of the renin-angiotensin system (RAS), and is obtained from angiotensin I (Ang I) by angiotensin converting enzyme (ACE), prolyl-endopeptidase (PEP) andneutral-endopeptidase (NEP) or angiotensin II (AngII) by angiotensin converting enzyme 2 (ACE2), PEP and prolyl-carboxypeptidase (PCP). Significantly, it displays weak affinity for the classic angiotensin type 1 (AT1) and type 2 (AT2) receptors. Recent experiments have identified the G-protein-coupled receptor (MAS), which might mediate the diverse biological functions of Ang1–7. It has been also demonstrated that a wide-ranging interaction between MAS1 and the AT1 and AT2 receptors may exist^1,2^.

AngII, a key factor in RAS, induces cell proliferation by activating the AT1 receptor; however, stimulation of the AT2 receptor inhibits cell growth in different cell types. Both the AT1 and AT2 receptors are present in tumors and in most cases, they are up-regulated. While AT2 and AT1 receptor expression is significantly elevated in prostate tumor tissue, prostate cancer is one of the few malignancies in which AT2 expression is known to be down-regulated in high-grade tumors^2^.

The molecular mechanisms regulating the level and activity of MAS1 in cancerous tissues remain poorly understood. Bernardi *et al*.^3^ found that *MAS1* was significantly up-regulated in colorectal carcinoma as compared to non-neoplastic colon mucosal tissue^3^. In contrast, Luo *et al*.^4^ noted that ductal carcinoma tissue *in situ* expressed MAS1 at high levels, but significantly lower as compared to non-cancerous tissues. Nevertheless, the observed decrease in MAS1 levels in breast cancer was associated with tumor growth, lymph node metastasis, and increased tumor grade as well as increased MIB-1 proliferation index and epidermal growth factor receptor (*EGFR*) and tyrosine-protein kinase erbB-2 receptor (*HER2*) gene expression^4^. Similarly, human osteosarcoma cell lines U-2 OS and MNNG-HOS with *MAS1* knockdown showed increased proliferation activity as compared to osteosarcoma cells non-treated siRNA^5^.

There are relatively few preclinical studies as well as clinical trials which show the antitumor activity of Ang1–7^6^. However, growing body of research suggests that the Ang1–7/MAS axis has anti-proliferative and anti-angiogenic effects on various types of cancer, including prostate cancer. Menon *et al*.^7^ evidenced that Ang1–7 inhibits cell proliferation and reduces tumor volume in the human lung tumor xenograft model^7^. Similarly, Pei *et al*.^8^ and Krishnan *et al*.^9^ found Ang1–7 to have anti-angiogenic properties and in consequence inhibit cancer cell invasion and metastasis^8,9^. A recent meta-analysis has reported association between *ACE* gene polymorphism and prostate cancer risk but only for the Asians and Latino population^10^.

In contrast to previous results, it has been also found that the ACE2/Ang1–7/MAS axis may promote the migration and invasion of renal cell carcinoma^11^ and mediate the AngII-induced epithelial-mesenchymal transition (EMT) in tubule cells^12^. These opposing results indicate that Ang1–7 plays pleiotropic roles in cancerogenesis and a complex network of interrelations might exist between the various elements of the local RAS.

## Materials and Methods

### Reagents

Ang1–7 (H-1715) was purchased from Bachem. The angiotensin receptor blockers losartan (AT1 antagonist), PD 123319 (AT2 antagonist), A779 (AT1–7/MAS antagonist) and HIF142 (AT4/IRAP antagonist) were obtained from TOCRIS Bioscience. For all experiments, heptapeptide was used at a final concentration of 1 nM, and inhibitors at a concentration of 1000 nM. This concentration was selected on the basis of earlier research work and literature data. Medium containing the mentioned above compounds was changed every 24 h. Unless otherwise specified, the medium and other culture supplements were purchased from Gibco; Thermo Fisher Scientific, Inc.

### Cell lines and culture conditions

The research was conducted on three stable cell models of prostate cancer: LNCaP cell line from DSMZ (Deutsche Sammlung von Mikroorganismen und Zellkulturen GmbH), DU-145 cell line from ATCC (American Type Culture Collection) and PC3 cell line from ECACC (90112714; European Collection of Authenticated Cell Cultures). Cell lines authenticity were confirmed by short-tandem repeat (STR) DNA profiling (LGC Standards Cell Line Authentication Service, Germany; 2014). The androgen-sensitive LNCaP cell line is a model of early, low invasiveness prostate cancer. The androgen-irresponsive DU-145 and PC3 cell lines were models of subsequent malignant stages of prostate cancer. The cells were passaged at least twice after thawing from liquid nitrogen and cultured in a humidified atmosphere at 37 °C with 5% CO_2_ in RPMI 1640 (LNCaP, PC3) or DMEM medium (PC3). In addition, standard supplements were used: 10% heat-inactivated Fetal Bovine Serum (FBS), 1 mM Sodium Pyruvate, 10 mM HEPES buffer and antibiotics (penicillin 50 U/ml; streptomycin 50 mg/ml; neomycin 100 mg/ml).

### Cell viability assay (MTT assay)

Ang1–7 was added to the cell culture medium at concentration 1 nM. Four hours prior to the end of the incubation period (48 hours), a MTT working solution at a final concentration of 0.5 mg/ml was added to each well. The formazan crystals formed by viable cells were dissolved in 10% sodium deodecyl sulfate (SDS) solution in 0.01 M HCl. Absorbance was measured at 570 nm using a microplate reader (BioTek). Cell viability (% of control) was calculated in relation to untreated cells.

### Cell proliferation assay

The changes in cell proliferation after Ang1–7 treatment (1 nM; 48 hours) were determined using a BrdU Cell Proliferation Assay (Merck Millipore) according to the manufacturer’s instructions. The principle of this non-isotopic immunoassay is that bromodeoxyuridine (BrdU) is incorporated as analog of the nucleotide thymidine into nuclear DNA, and this can serve as a label that can be detected using antibody probes. The colored reaction product was quantified using a BioTek microplate reader at a wavelength of 450 nm. Cell proliferation (% of control) was calculated in relation to untreated cells.

### Cell cycle assay

After 48 hours of incubation time with Ang1–7 (1 nM), alone or in combination with angiotensin receptor inhibitors (1000 nM), the prostate cancer cells were collected by traditional trypsin/EDTA treatment. The percentages of cells in the G0/G1, S, G2/M phases of cell cycle was determined using a Muse™ Millipore Cell Cycle Kit (Merck Millipore) according to the manufacturer’s instructions. The assay utilizes propidium iodide (PI)-based staining of DNA content to defined and measure the percentage of cells in the various cell cycle phases.

### Cell adhesion assay

The prostate cancer cells were exposed to Ang1–7 at a concentration of 1 nM while the controls were grown in standard cell culture medium. After 48 hours of incubation time, the cells were collected by traditional trypsin/EDTA treatment. The cell suspension in FBS-free medium (1 × 10^6^/ml) was added to extracellular matrix (ECM)-coated wells (collagen I, collagen IV, fibronectin and gelatin; BD Biosciences). The non-adherent cells were removed by washing three times with PBS after 90 minutes incubation period at 37 °C in a humidified atmosphere of 95% air −5% CO_2_. The samples were fixed and stained with a 0.1% solution of crystal violet (Sigma Aldrich) in 25% ethanol. Finally, cells were rinsed in water and dissolved in 10% acetic acid. Absorbance was measured at 570 nm on a BioTek microplate reader. The data were presented as a percentage of the untreated control.

### Soft agar colony formation assay

The assay was performed in a 6-well plate coated with 0.9% agar in growth medium. The cell suspension was perpetrated at a final concentration of 1 × 10^4^ cells/ml of 0.3% agarose in complete medium and were added to the top of the solidified base layer agar. The cells were incubated at 37˚C in 5% CO_2_, and were treated with another dose of Ang1–7 (1 nM) every two to three days. After 11–14 days, the colonies were stained with 0.05% crystal violet for at least 30 minutes. The results were recorded and documented by scanning the plates [Suplementary Material]. The number of colonies in the semisolid medium and total area of colonization were counted using ImageJ software (http://imagej.nih.gov/ij).

### Wound healing assay

All cell lines were seeded on 6-well plates and cultured until 90–100% confluence at 37 °C and 5% CO_2_. A regular scratch was performed using a sterile pipette tip, and the monolayer was washed twice with 2–3 ml of sterile PBS to discard cell debris. Ang1–7 (1 nM) was added to the culture medium every 24 hours for two days. Wound assessment was performed by photographing of the same region of the well after: 0 hours, 24 hours and 48 hours [Suplementary Material]. The area of the wounded surface and its closure was calculated by ImageJ software (http://imagej.nih.gov/ij). The test was performed in four independent replicates, then each control sample was compared to each test sample.

### Transwell migration/invasion chamber assay

The transwell migration/invasion assays were performed as described previously^13^. All cell lines were incubated with Ang1–7 (1 nM) on 6-well plates for 48 hours. The cells were then added to porous inserts of 8 µm pores in polyethylene terephthalate membranes at a density of 1 × 10^−6^ cells/ml in 300 µl of serum-free medium. In the case of cell invasiveness the test membrane was coated with Matrigel (Thermo Fischer Scientific, Inc.): a mixture of ECM proteins. The lower part of the well was filled with 600 µl of medium supplemented with 10% FBS. After 24 hours of incubation at standard conditions (37 °C, 5% CO_2_), any cells that had not migrated through the membrane were mechanically removed. The cells that successfully migrated through the membrane were stained (0.1% solution of crystal violet in 25% ethanol) and dissolved (10% acetic acid). The results were measured by spectrophotometric analysis at 570 nm (BioTek). Data was expressed in relation to untreated controls and expressed as the percentage difference of Ang1–7 treated cells.

### Gelatin zymography assay

The prostate cancer cells were plated onto 6-well plates and incubated until 80–90% confluence. Then the medium was changed to fresh serum-free medium with or without Ang1–7 at a concentration of 1 nM. After 24 hours of incubation, the culture medium was collected. The protein concentration in the supernatant was measured using a Qubit® Protein Assay kit (Invitrogen™ Thermo Fisher Scientific, Inc.). Protein extracts (10 µg) were separated by 10% SDS-PAGE gel, supplemented with gelatin (2 mg/ml). First, the gel was incubated in a developing buffer (0.5 M Tris-HCl, 2 M NaCl, 50 mM CaCl_2_, pH 7.5) and then with Coomassie Brilliant Blue R-250 staining buffer. Following this, a discoloring buffer comprising methanol: acetic acid: water (3: 1: 6) was added to identify clear bands on the dark background, as previously described^13^. The gelatinolytic activity of enzymes in samples was further evaluated based on the band area using ImageJ software. Images of gels are incuded in the Suplementary Material section.

### RT-qPCR (quantitative reverse transcription PCR)

The prostate cancer cells were exposed to Ang1–7 at a concentration of 1 nM for 48 hours. Total RNA was extracted from the cells using TRIzol reagent (Thermo Fisher Scientific, Inc.) and purified with the standard phenol-chloroform method. The concentration of recovered RNA and its purity was determined by BioDrop μLITE. Reverse transcriptase was used to synthesize cDNA for further RT-qPCR analysis. Amplification reactions were performed with a LightCycler 480 Real-Time PCR System (Roche Diagnostics) and EvaGreen PCR master mix (IMMUNIQ) as described previously^14^. Quantitative data from gene expression experiments was normalized to the expression of two housekeeping genes: *H3F3A* (homo sapiens H3 histone, family 3A) and *RPLPO* (ribosomal protein lateral stalk subunit P0). The Universal Human Reference RNA (Stratagene), composed of total RNA from 10 human cell lines, was used as a calibrator for each reaction. The primers and reaction conditions were presented previously^15^. All the reactions were run in triplicate, including no-template controls. Differences in gene expression were calculated by REST-MCS beta software version 2 (2006) (www.gene-quantification.info).

## Results

### Changes in cell viability and proliferation after Ang1–7 treatment

**Figure 1:**
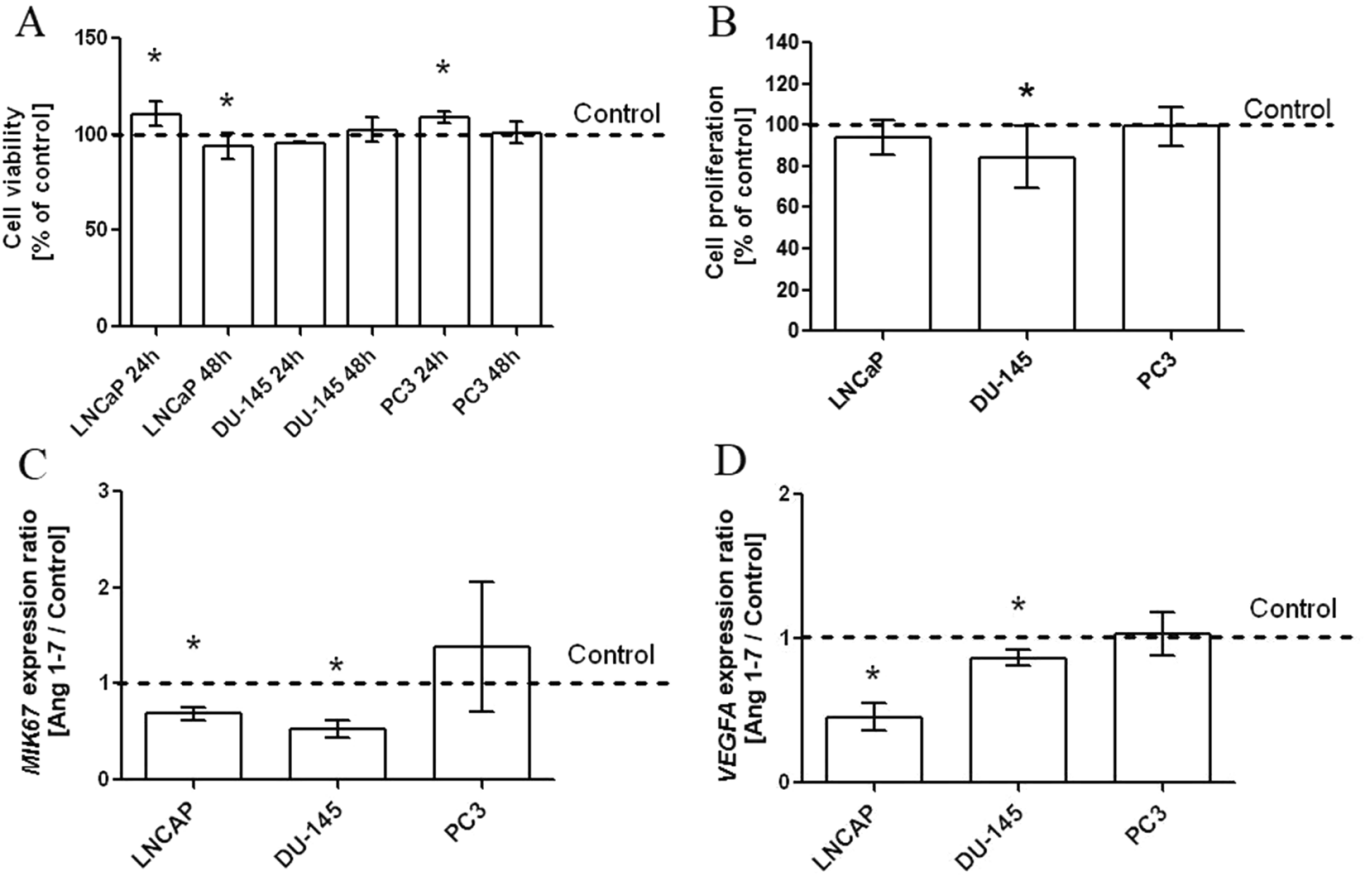
Cell viability and proliferation of prostate cancer cells (LNCaP, DU-145, PC3) after exposure to Angiotensin 1–7 (mean ± SD; Tukey’s test: *p < 0.05). Panel (A) shows results of BrdU Assay and MTT Assay. Panel (B) presents results of expression of *MKI67* and *VEGFA* genes.

Ang1–7 (1 nM) increased the cellular metabolic activity of LNCaP and PC3 cells but only during the short incubation (24 h): those effects were overcome by prolongation of exposure with heptapeptide (Fig. 1A). In the case of the androgen-dependent prostate cancer line, inhibition of both cell viability and proliferation were observed after 48-hour incubation with Ang1–7 (Fig. 1B). However, statistically significant differences in the level of incorporation of exogenous BrdU into genomic DNA were observed only between the control and the experimental DU-145 prostate cancer cells. It is worth emphasizing that RT-qPCR showed that Ang1–7 can reduce the level of expression of the proliferation marker in LNCaP and DU-145 cells. These two lines also demonstrated a decrease in the mRNA level of the angiogenesis marker after 48-hour incubation with heptapeptide. No statistically significant differences were observed between *MKI67* and *VEGFA* level in either the induced or the control PC3 cells (Fig. 1C).

### Changes in the percentage of cells in the G0/G1, S, G2/M phases of the cell cycle after treatment with Ang1–7 alone

**Figure 2:**
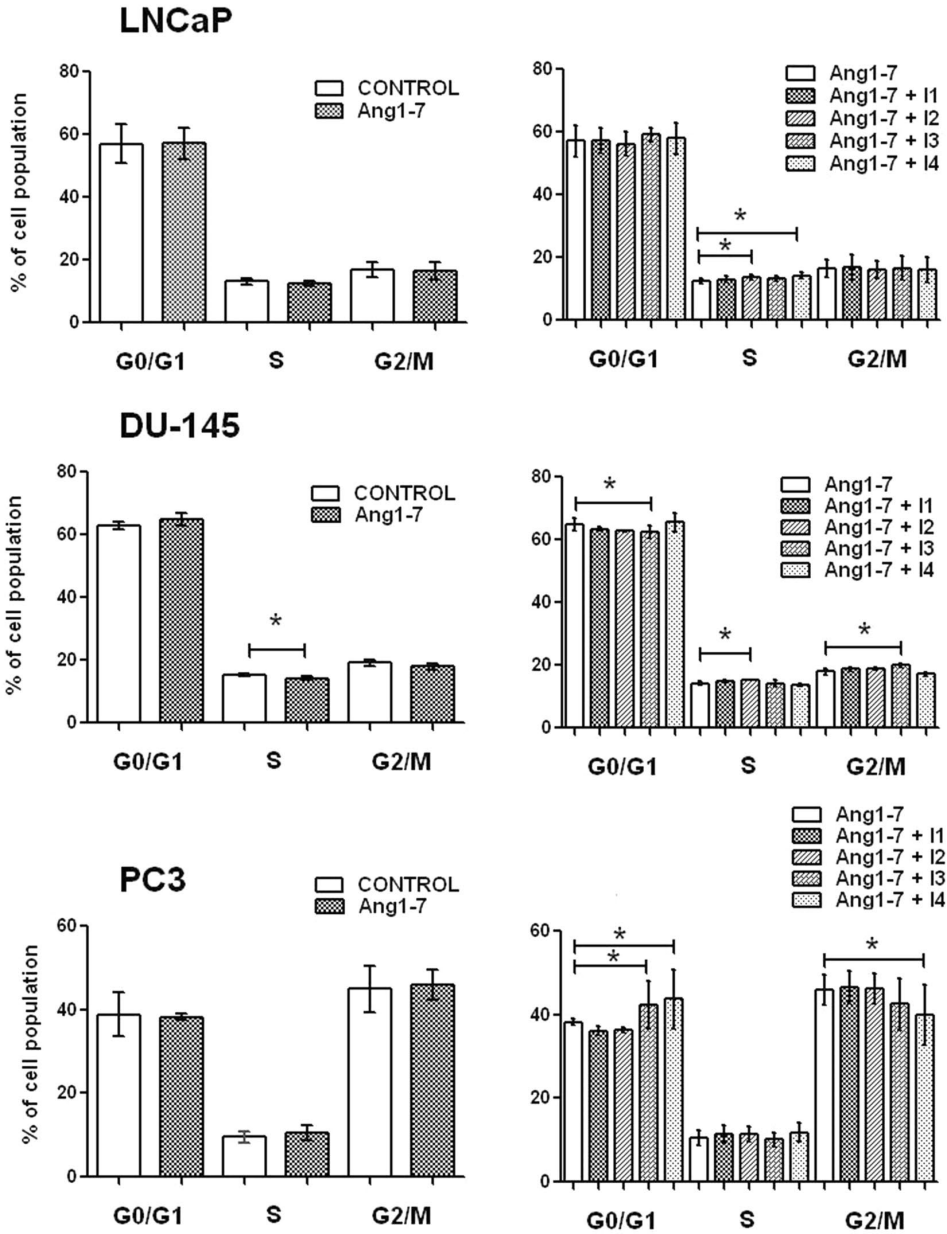
The percentage of prostate cancer cells (LNCaP, DU-145, PC3) in the G0/G1, S, G2/M phases of cell cycle following exposure to Angiotensin 1–7 alone or in combination with angiotensin receptor inhibitors (I1: AT1 inhibitor - losartan; I2: AT2 inhibitor - PD123319; I3: AT1–7/MAS - A779; I4 – AT4/IRAP - HFI142) (mean ± SD; Tukey’s test: *p < 0.05).

Muse Cell Cycle Assay revealed the presence of significant changes in cell cycle distribution only in the DU-145 cell line. Ang1–7 decreased the proportion of cells in the S phase population as compared to untreated cells. LNCaP cells also demonstrated a downward trend in the part of the cell cycle in which DNA is replicated; however, the opposite situation was recorded in the PC3 cell line. In the case of LNCaP and DU-145 cells, AT1–7/MAS and AT2 receptors were found to be involved in Ang1–7 mediation of the signal. However, in the androgen-independent PC3 line, significant changes were observed between the use of Ang1–7 alone and in combination with inhibitors of AT1–7/MAS and AT4/IRAP receptors (Fig. 2).

### Changes in the ability to adhere to the ECM proteins and anchorage-independent cell growth after Ang1–7 treatment

**Figure 3:**
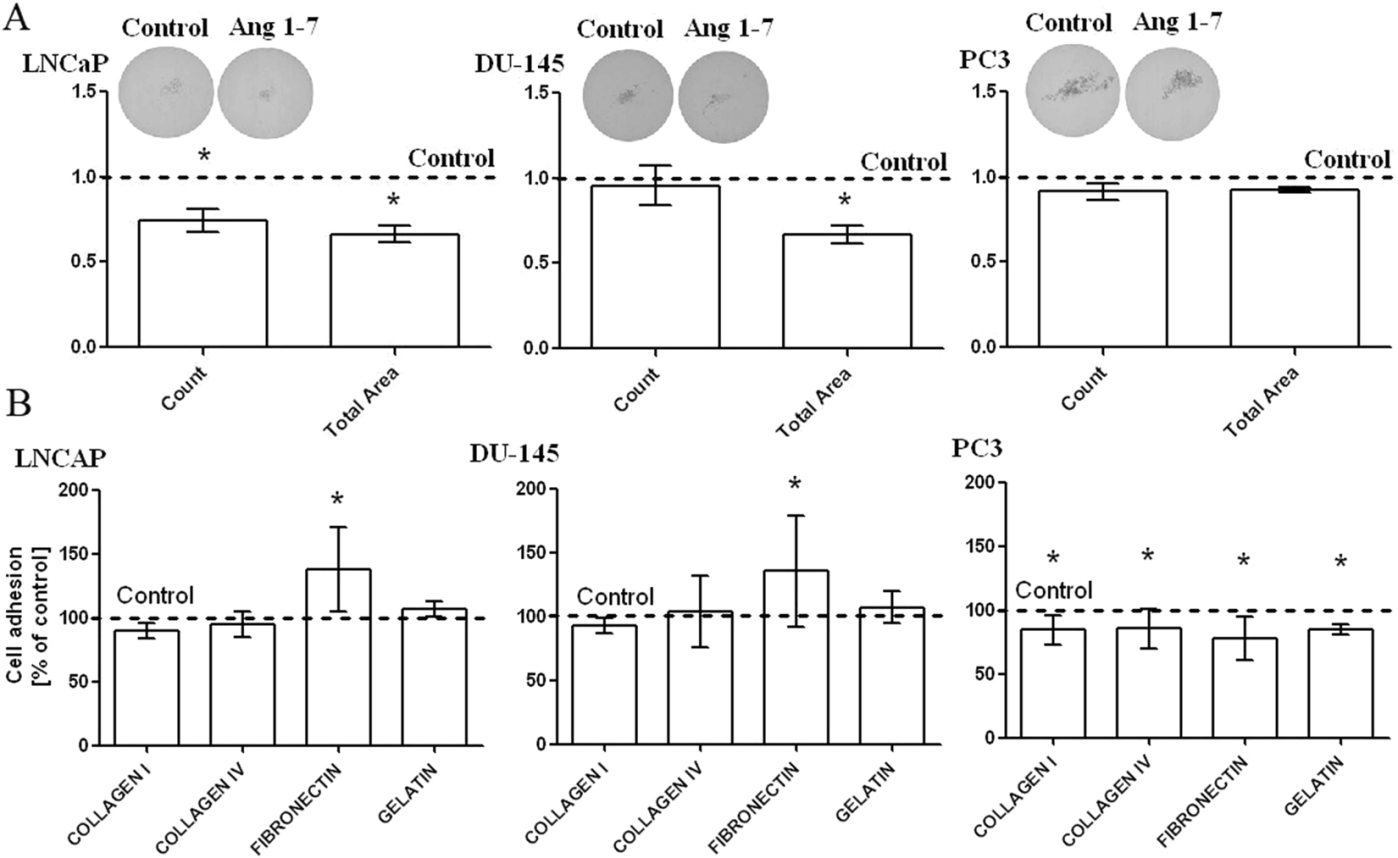
The ability of adhesion and anchorage-independent cell growth after Ang1–7 treatment Panel (A) shows results of Soft Agar Colony Formation Assay (mean ± SEM; Tukey’s test: *p < 0.05). Panel (B) presents results of ECM Cell Adhesion Assay (mean ± SD; Tukey’s test: *p < 0.05).

After 48-hour incubation with Ang1–7 significant differences were found between LNCaP and DU-145 cells ability to adhere to fibronectin. The number of adherent cells increased by over 130% compared to the control sample. On the other hand, treatment with the heptapeptide resulted in lower adhesion of PC3 cells to all tested extracellular matrix proteins, i.e. both types of collagen, fibronectin and gelatin (Fig. 3B).

As shown in Fig. 3, Ang1–7 decreased the growth of the LNCaP cell line in soft agar. A significant reductions were seen in the size and number of colonies. In the case of DU-145, while the number of colonies remained unchanged, the area occupied by the colonies increased. Ang1–7 has no influence on the anchorage-independent growth of PC3 cell line (Fig. 3A).

### Changes in migration and invasion after Ang1–7 treatment

**Figure 4:**
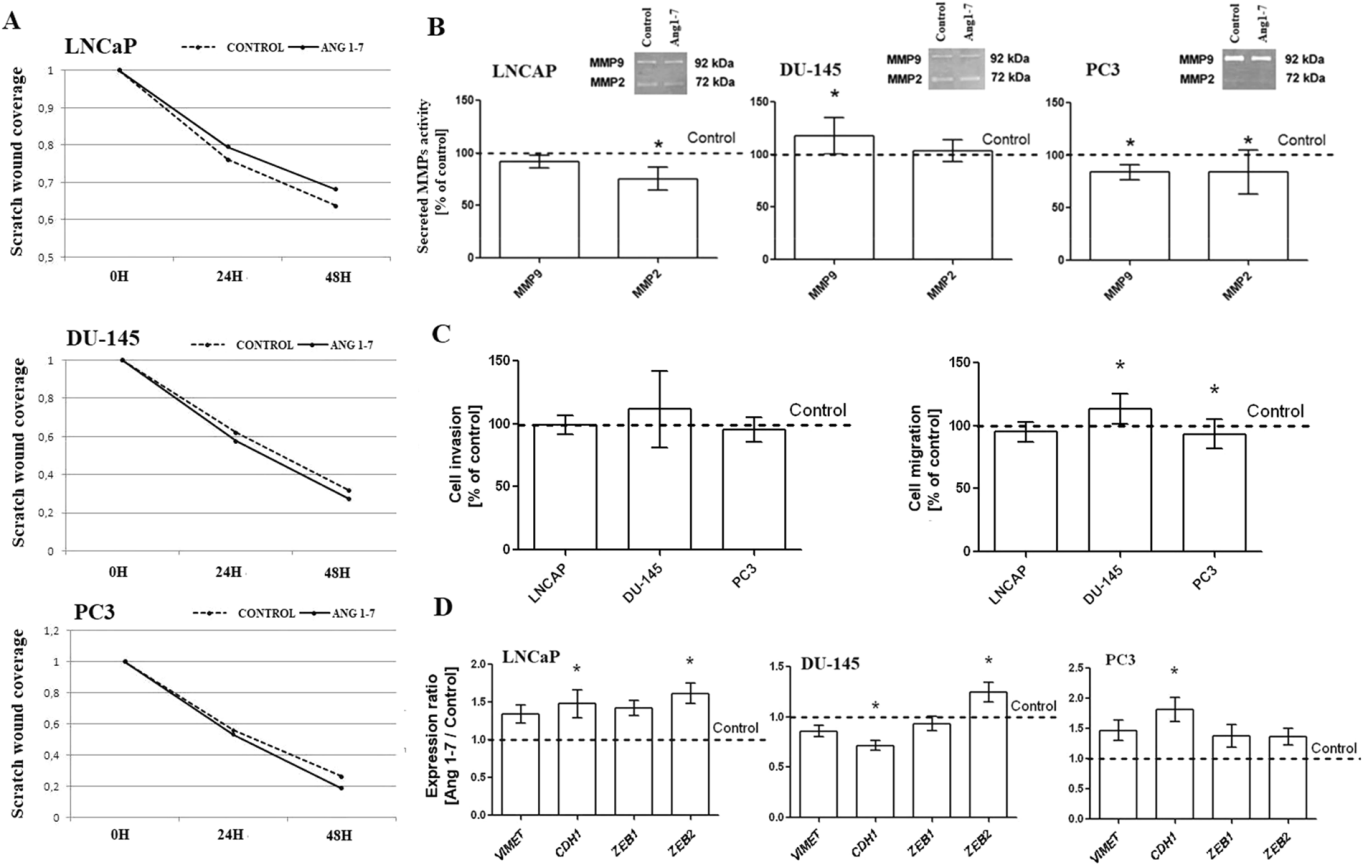
Changes in the migration and invasion potential of the androgen-dependent (LNCaP) and androgen-independent prostate cancer cell lines (DU-145 and PC3) after exposure to Angiotensin 1–7. The left panel (A) presents the results of the wound healing assay. Panel (B) shows results of Gelatin Zymography to detect matrix metalloproteinase (MMP) activity The results in transwell migration/invasion assay are visible on the panel (C) (mean ± SD; Tukey’s test: *p < 0.05). Panel (D) present the expression of the EMT markers (mean ± SEM; Tukey’s test: *p < 0.05).

No significant changes in the prostate cancer cell invasion were found after 48 hours of incubation with Ang1–7. Similarly, the wound healing assay did not reveal any important changes between control and treated cells (Fig. 4A). However, the LNCaP and PC3 lines demonstrated a decreased ability to migrate through 8 µm pore size inserts, while the mobility of the DU-145 cells increased (Fig. 4C). The result for the androgen-dependent cell line was not statistically significant. In the case of the DU-145 cell line, the expression of the E-cadherin gene (*CDH1*) was also found to be decreased. The other two prostate cancer lines showed an increase in the expression of *CDH1*. In addition, LNCaP and DU-145 cells presented elevated zinc finger E-box binding homeobox 2 (*ZEB2*) expression (Fig. 4D).

The level of two matrix metalloproteinases (MMPs) were determined via gelatin zymography. The samples contained two prominent gelatinolytic bands corresponding to monomeric pro-MMP-9 and pro-MMP-2. The strongest bands were the single 92 kDa band in PC3 cells, and the 72 kDa band in DU-145 cells. Ang1–7 caused a decrease in Gelatinase A in LNCaP and both of the MMPs in PC3 cells, but also increased Gelatinase B in DU-145 cell line (Fig. 4B).

### Changes in gene (mRNA) expression level after Ang1–7 treatment

**Figure 5:**
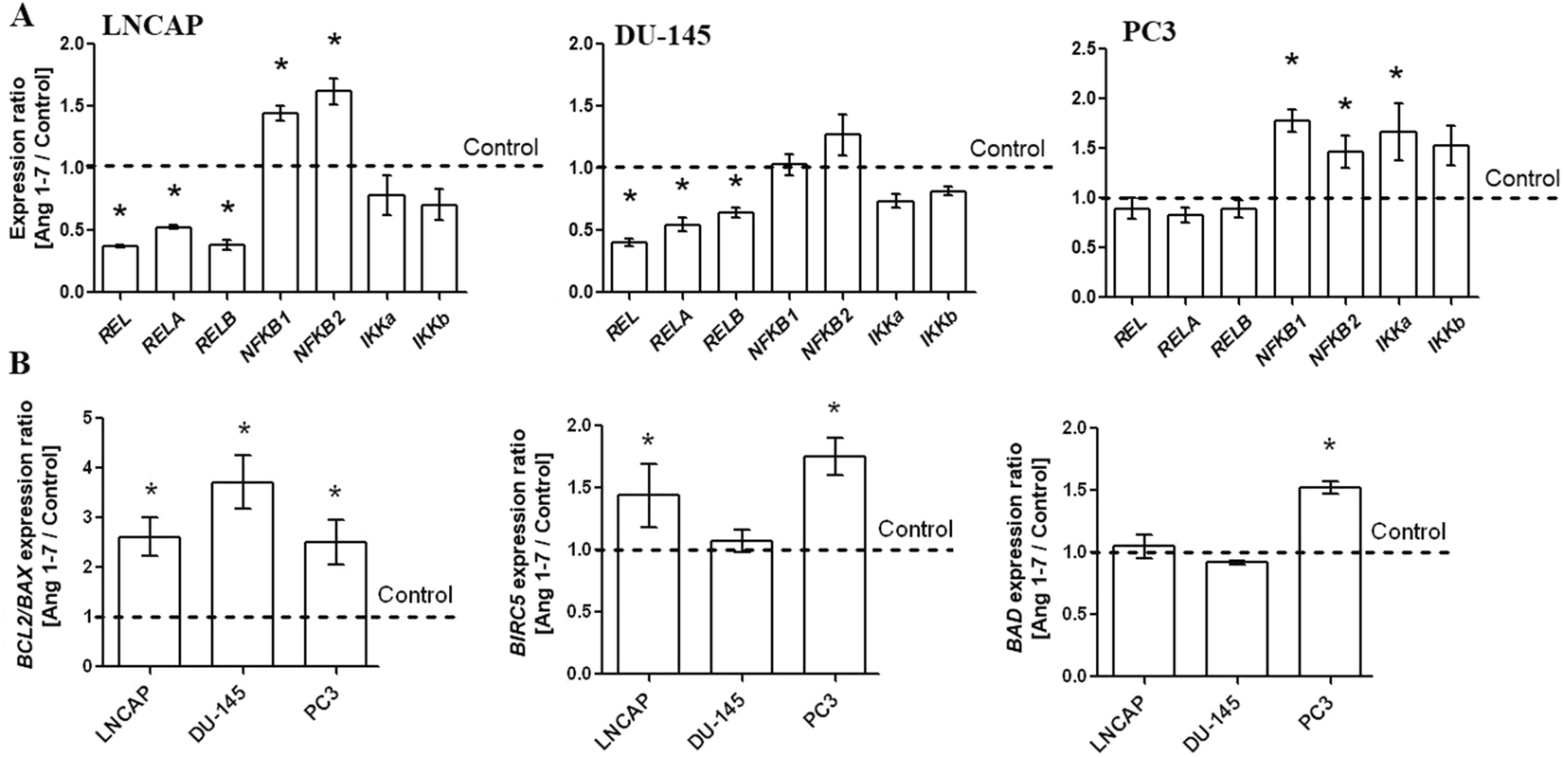
Expression of *NFKB* and *IKK* family genes (**A**) and expression of anti- and pro-apoptotic members (**B**) in the androgen-dependent (LNCaP) and androgen-independent prostate cancer cell lines (DU-145 and PC3) following exposure to Angiotensin 1–7 (mean ± SEM; Tukey’s test: *p < 0.05).

*NFKB1* (nuclear factor-κB subunit 1) and *NFKB2* (nuclear factor-κB subunit 2) gene expression was up-regulated by Ang1–7 in all prostate cancer cells, but this increase was not statistically significant for the DU-145 line. On the other hand, the expression of the *REL* (REL proto-oncogene nuclear factor-κB subunit), *RELA* (RELA proto-oncogene nuclear factor-κB subunit) and *RELB* (RELB proto-oncogene nuclear factor-κB subunit) genes were down-regulated by Ang1–7 in all prostate cancer cells; however, the decrease was not statistically significant for the PC3 line. In general, IκB kinase (*IKK*) family genes expression did not undergo significant change after incubation with peptide tested. However, we have observed a clear downward trend for the expression of *IKK* in LNCaP and DU-145 lines and an upward trend for PC3 cells which was significant for *IKKa* (Fig. 5A).

The changes in gene expression of anti- and pro-apoptotic members after exposure to Ang1–7 are summarized in Fig. 5B. Decreased *BAX* (BCL2 associated X, apoptosis regulator) expression and increased *BCL2* (BCL2, apoptosis regulator) expression were observed in all prostate cancer cells (date not shown). The most accentuated growth ratio of *BCL2*/*BAX* was found in DU-145 cells. However, this cell line was the only one not to show upregulated *BIRC5* (baculoviral inhibitor of apoptosis repeat-containing 5/survivin) expression after Ang1–7 treatment. The level of *BAD* (BCL2 associated agonist of cell death) was also found to be elevated in the PC3 androgen-independent cell line (Fig. 5B).

**Figure 6:**
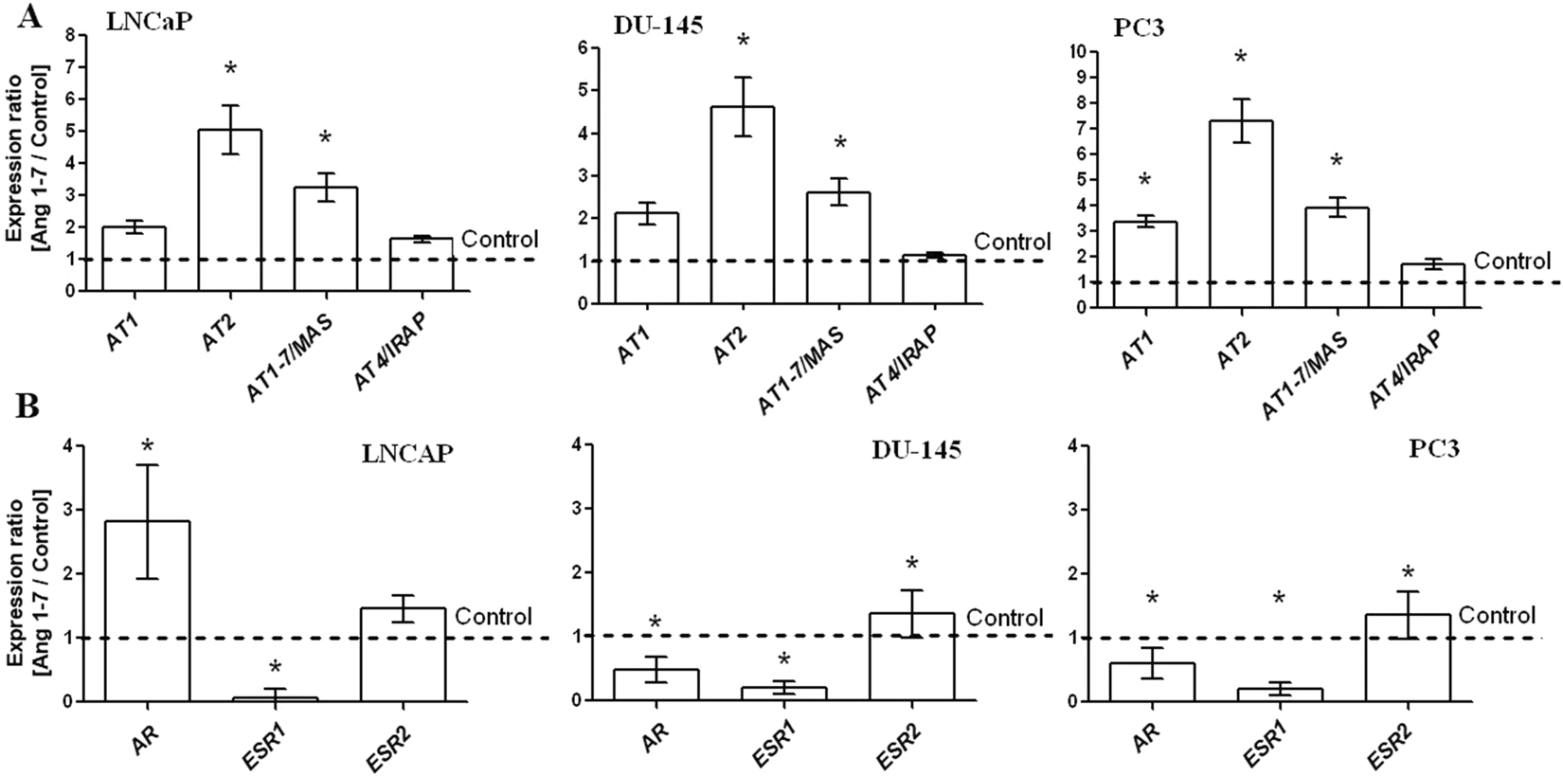
Expression of angiotensin receptor genes and expression of sex steroid hormone receptors in the androgen-dependent (LNCaP) and androgen-independent prostate cancer cell lines (DU-145 and PC3) following exposure to Angiotensin 1–7 (mean ± SEM; Tukey’s test: *p < 0.05).

RT-qPCR results showed that angiotensin receptors are expressed in all of the tested prostate cancer lines. The following trends were observed for angiotensin receptor level with regard to type of prostate cancer cell line: *AT1*: PC3 > LNCaP > DU-145; *AT2*: LNCaP > DU-145 > PC3; *AT1*–*7*/*MAS*: DU-145 > LNCaP > PC3; *AT4*/*IRAP*: PC3 > LNCaP > DU-145. Ang1–7 can change mRNA expression level not only *AT1*–*7*/*MAS* receptor, but also the classic angiotensin receptors *AT1* and *AT2*. However, only PC3 cells demonstrated significantly greater *AT1* receptor expression. The expression of the *AT4*/*IRAP* receptor was not influenced by Ang1–7 (Fig. 6A).

The androgen receptor (*AR*) was expressed in both androgen-dependent and -independent prostate cancer lines. The expression of *AR* in DU-145 and PC3 cells was many times lower than in LNCaP cells (LNCaP > DU-145 > PC3). After exposure to Ang1–7 a significant up-regulation of *AR* expression was observed in androgen-dependent prostate cancer cells while a significant down-regulation was seen in the androgen-independent cells. The following trend in estrogen receptors (*ESRs*) expression levels was observed with regard to prostate cancer cell line: PC3 > DU-145 > LNCaP. It is worth emphasizing that estrogen receptor 2 (*ESR2*/*ERβ*) was highly expressed, while estrogen receptor 1 (*ESR1*/*ERα*) had a relatively low level of expression or was almost undetectable in prostate cancer lines. A 48-hour incubation with this heptapeptide resulted in significantly decreased expression of *ESR1* and a noticeable increase in the mRNA level of *ESR2* in prostate cancer cells (Fig. 6B).

## Discussion

The latest research adds further evidence that Ang1–7 is a RAS peptide with anti-proliferative properties, which can inhibit various types of tumor, in a time- and dose-dependent manner, through arresting cell division. Previously, Gallagher *et al*.^16^ found that Ang1–7 markedly inhibited the *in vitro* DNA synthesis of lung cancer cell growth^16^. Similarly, Liu *et al*.^17^ observed that Ang1–7 treatment dose-dependently reduced the growth of murine hepatocellular carcinoma *in vivo*^17^. Our findings indicate that Ang1–7 (1 nM; 48 h) can effectively reduce cell proliferation only in DU-145 cells; however, a significant decrease in M*KI67* expression was also observed in the case of an androgen-dependent prostate cancer cell line. The Ki-67 antigen is present during all active phases of the cell cycle, such as G1, S, G2 and mitosis, but is absent in quiescent cells (G0), which makes this non-histone nuclear protein a sensitive marker of cell proliferation^18^. Furthermore, Verma *et al*.^19^ present a statistically significant correlation between Ki-67 positivity and Gleason’s grade of prostatic carcinoma^19^.

Our findings suggest that the PC3 line may be unresponsive to such low doses of Ang1–7 in relation to cell division. Later parts of the discussion associate these findings also with the increased *BAD* expression in this cell line. In contrast, Krishnan *et al*.^20^ reported that Ang1–7 attenuates prostate cancer cell growth for both the DU-145 and PC3 cell lines; however, it should be emphasized that a much higher peptide concentration (100 nM) and a longer incubation (6–10 days) were used. A clear differences between the numbers of control and treated cells were observed much later in the PC3 line than the DU-145 line^20^.

To understand the functional role of Angiotensins in cancer development, it is necessary to study the expression and activation of angiotensin receptors. The anti-proliferative and anti-angiogenic effects of the Ang1–7/MAS axis in cancer have been evaluated^21,22^. Although Ang1–7 binds weakly to AT1 and AT2, the MAS receptor has been shown to interact functionally with classic angiotensin receptors. Some studies have reported that Ang1–7 can regulate not only the mRNA levels of *MAS*, but also those of the *AT1* and *AT2* receptors. Liu *et al*.^17^ noted that *AT1* expression was significantly decreased, whereas the levels of *AT2* and *MAS* receptors were found to be increased by Ang1–7 treatment in both *in vivo* and *in vitro* studies^17^.

The three prostate cancer cell lines used in the present study are characterized by individual profiles of angiotensin receptors. The PC3 cells demonstrated highest *AT1* expression level, while LNCaP demonstrated the highest *AT2* level. The expression of the *AT1*–*7*/*MAS* receptor was predominated in the DU-145 cell line, and *AT4*/*IRAP* in the PC3 cells (data not shown). Similarly to Chinese scientists^17^, our present findings indicate an increase in the expression of *AT2* and *MAS* receptors after incubation with Ang1–7. No down-regulation in *AT1* expression was observed in any prostate cancer line whereas a significant increase in expression was seen in the PC3 line. An earlier study presented that Ang1–7 can increase the protein level of the AT1 receptor in androgen-independent prostate cancer cells, and that this effect was attenuated by 17β-estradiol, not by testosterone^23^. Although the current study also evaluated the effect of Ang1–7 on the *AT4*/*IRAP* receptor, its mRNA level was found to remain unchanged.

Interestingly, Liu *et al*.^17^ showed that the impact of Ang1–7 on the expression of AT1 and AT2 receptors was partially blocked by co-administration of A779 or PD123319, whereas *MAS* mRNA level was partially reversed by the MAS receptor antagonist, but not AT2 inhibitor. Furthermore, the beneficial effects of Ang1–7 on H22 cell viability and proliferation, as well as tumor growth, were offset by A779 or PD123319^17^. Our findings also confirmed that not only A779 inhibitor, but also PD123319 can reverse the action of Ang1–7. It is important to note that this heptapeptide may also act through AT2 receptor, which can open an extensive discussion on its nature and physiological properties.

Cancerogenesis is widely known to be a dynamic process that depends on a huge number of factors and multiple pathways such as IκB kinase (IKK)/nuclear factor κB (NF-κB) pathway^24–27^. Jiang *et al*.^25^ report that part of the protective effect of Ang1–7 in stroke was associated with its modulatory effects on NF-kB and inflammation^25^. The NF-κB family of transcription factors plays an essential role in cancer development and progression, cell proliferation and differentiation, and the regulation of cell survival and apoptosis. In humans, the NF-κB family is composed of five related transcription factors: NF-κB1 (p105/p50), NF-κB2 (p100/p52), RelA (p65), RelB (p66), and c-Rel (p75) subunits, encoded by the *NFKB1*, *NFKB2*, *RELA*, *RELB*, and *REL* genes, respectively. The transcription factor NF-κB is constitutively activated during several human malignancies, including prostate cancer^26,27^. Shukla *et al*.^28^ suggest that RELA/p65 (but not NF-κB1/p50) is constitutively activated in human prostate adenocarcinoma^28^. The present study demonstrates that Ang1–7 alters the mRNA expression of NF-κB family members in prostate cancer cell lines. *NFKB1* (chromosome 4q24) and *NFKB2* (chromosome 10q24) were upregulated by Ang1–7 whereas *RELA* (chromosome 11q13), *RELB* (chromosome 11q13) and *REL* (chromosome 12p12) can be downregulated. In the case of androgen-sensitive prostate cancer cells, only one of two variants were observed: i.e. upregulated (in PC3) or downregulated *NFKB* expression (in DU-145). Our previous study found that AngII also modulated the mRNA expression of NF-κB family members however, Ang1–7 and AngII have clearly the opposite effect on the level of *NFKBs* expression: AngII significantly decreased the expression of *NFKB1* mRNA in all prostate cancer cells while *NFKB2* did so only in the PC3 cell line. On the other hand, this octapeptide increased the level of *RELA* expression in LNCaP, and the levels of PC3 and *REL* in LNCaP and DU-145 cells^14^. A numerous studies confirmed that Ang1–7 frequently acts as a functional antagonist of Ang II.

High nuclear levels of RelB and RelA have been detected in human prostate cancer and are related to a poor prognosis in patients^29–32^. The level of RelA and c-Rel in prostate cancer tissues correlated with their Gleason’s score and were higher than those of biopsy samples containing benign epithelium^33^. Similarly, high constitutive nuclear levels of RelB have been noticed in human prostate cancer specimens with high Gleason scores^32^. Unexpectedly, levels of prostate specific antigen (PSA) inversely correlate to reduced levels of both RelA and RelB^32,34^. Mukhopadhyay *et al*.^35^ also showed that overexpression of c-Rel downregulated the promoter activity of PSA in LNCaP cells^35^. In contrast, Shukla *et al*.^28^ did not observe any significant correlation between serum PSA levels and NF-κB expression in androgen-insensitive human prostate carcinoma PC3 cells^28^.

Interestingly, Xu *et al*.^34^ observed that the tumorigenicity of androgen-independent PC3 cells was reduced by *RELB* knockdown while LNCaP cell tumorigenicity was enhanced following *RELB* overexpression. Furthermore RelB contributes to radioresistance of PCa^34^. In our study, Ang1–7 decreased the mRNA level of *RELA*, *RELB* and *REL* gene, thus Ang1–7 might potentially contributes to the suppression of prostate cancer.

The two IĸB serine-threonine kinases: IKKa and IKKb are critical in activating the NF-ĸB pathway^24,26–29^. In our study Ang1–7 caused a downward trend of expression level of *IKK*a and *IKKb* in the LNCaP and DU-145 lines and an upward trend in PC3 cells. Immunohistochemistry analysis of human prostate cancer tissue microarrays (TMA) demonstrate that phosphorylation of IKKa/b within its activation loop gradually increases in low to higher stage tumors as compared with normal tissue^36^. Mahato *et al*.^37^ suggested that IKKa may regulate invasion and metastasis of prostate cancer cells by the Ras-MAPK and PI3K-Akt pathways^37^. In turn, Zang *et al*.^36^ observed that IKKb activity is regulated by Akt, mTORC1, and IKKa^36^. Increasingly, inhibitors IKKs are considered as a potentially useful strategy especially for treating hormone refractory prostate cancers. However, no potent and non-toxic IKKa/b inhibitor has been identified so far^24,36,37^.

A number of important genes for cancerogenesis are regulated by NF-κB, such as anti-apoptotic genes (*BIRC5* or *BCL2*), genes modulating epithelial-mesenchymal transition (*CDH1*, *ZEB*) and genes involved in tumor metastases (*MMPs* or *VEGF*)^26,27^. Shukla *et al*.^28^ suggest that increased *BCL2* expression correlates with DNA binding and nuclear localization of NF-κB/p65 in prostate cancer specimens^28^. In the present study, the expression ratio of *BCL2*/*BAX* was found to be significantly greater after Ang1–7 treatment in all prostate cancer cells. Furthermore, Ang1–7 incubation induced upregulation of survivin (*BIRC5*) expression in LNCaP and PC3 cells; survivin is a member of the family of apoptosis inhibitors (IAPs)^38^. Our results suggest that low concentrations of Ang1–7 (1 nM) may prevent the cells from apoptosis and might promote cell viability. This seems possible, especially at the initial stages of the heptapeptide activity: following treatment with Ang1–7, increased metabolic activity was observed in LNCaP and PC3 cells after 24-hours but not 48-hours. In addition, the androgen-dependent prostate cancer cell line demonstrated a significant decrease of cell viability after a prolonged period of incubation.

Increased expression of the apoptotic gene *BAD* was observed only in PC3 cells. It is possible that BAD provides a counterbalance for up-regulated levels of BCL2, which are known to slow proliferation^39^. Generally, BAD seems to be an appropriate modulator of BCL2/BCLXL due to the fact that its ability to form heterodimers depends on phosphorylation status^40^. Interestingly, BAD immunoexpression has been found to be higher in prostatic carcinomas than normal prostatic epithelium^41^, which raises the question of why a higher level of pro-apoptotic protein should be found in a prostate cancer cell. Smith *et al*.^40^ suggest that BAD plays a dual role in prostate cancer cells: the dephosphorylated form of this protein promotes apoptosis, while the phosphorylated form can stimulate cell proliferation and tumor growth^40^. In fact, only in the case of the PC3 line, which showed up-regulation of the *BAD* gene, no decrease of cell proliferation was observed after Ang1–7 treatment.

An unbalanced expression of MMPs and their inhibitors (TIMPs) is seen in prostate cancer tissue. The upregulation of MMP activity promotes a prostate progression cascade by modulation of cell proliferation, EMT process, invasion and angiogenesis. It is generally thought that MMP-2 and MMP-9 are more active in the advanced stages of prostate cancer, and are significantly associated with Gleason score^42^. Ni *et al*.^43^ found that Ang1–7 reduced the migratory and invasive abilities in A549 human lung adenocarcinoma cells by reducing the expression and activity of MMP-2 and MMP-9^43^. Cambados *et al*.^44^ postulated that Ang1–7 abolished Ang II induced stimulation of MMP-9 activity and VEGF expression in breast cancer cells (MDA-MB-231)^44^. On the other hand, it was noticed that Ang1–7 directly increased the activity of MMP-2 and MMP-9, decreasing the content of TIMP-1 and TIMP-2 in cardiac fibroblasts^45^.

Our findings confirm that Ang1–7 can also modulate the activity of both gelatinases A and B in prostate cancer cells, that MMP-9 activity was upregulated in DU-145 and downregulated in PC3, and that MMP-2 levels were suppressed in LNCaP and PC3 cells. Not surprisingly, it was found that DU-145 cells demonstrate greater migration and invasion potential after incubation with Ang1–7; however, LNCaP and PC3 cell lines demonstrated less cell movement after Ang1–7 treatment. However, it must be emphasized that not all results were statistically significant and unambiguous. The results of the examination of migration process via porous chambers are not fully coherent with the results of the wound healing assay. Although they examine the same effect, these two processes possess different mechanisms. The previous studies also give inconsistent results regarding Ang1–7 treatment: Ni *et al*.^43^ notes that the invasive abilities of lung carcinoma were indeed diminished, but the outcomes were strongly dosedependent^43^, while Zhang *et al*.^46^ found Ang1–7 to have no effect on the migration of smooth muscle cells, but that AngII was found to increase it^46^.

The MMPs are able to directly and indirectly induce the release of vascular endothelial growth factor (VEGF) by different types of cancer cells^47,48^. Surprisingly, the expression of *VEGFA* was found to be decreased after Ang1–7 treatment in LNCaP and DU-145 cells, but not in PC3. On the other hand, VEGF was shown to significantly reduce MMP-9 production in B-cell leukemia cells via STAT1 activation^49^. Furthermore, Shibata *et al*.^50^ reported that inhibition of NF-κB activity can decrease the expression of *VEGF* in breast cancer cells (MDA-MB-231)^50^. In the present study such downregulation of *VEGFA* mRNA was only observed in cell lines with lower levels of *RELA*, *RELB* and *REL*. Nevertheless, VEGF is clearly a key modulator of angiogenesis and hence Ang1–7 may inhibit the growth of new blood vessels from pre-existing vessels in prostate cancer.

The anti-angiogenic activities of Ang1–7 have been described previously. For example Krishnan *et al*.^9^ reported that Ang1–7 administration increased the soluble fraction of soluble fms-like tyrosine kinase-1 (sFlt-1), an antagonist of VEGF and placental growth factor (PlGF), resulting in the proliferation and angiogenesis of human prostate cancer xenografts^9^. Soto-Pantoja *et al*.^51^ noted that Ang1–7 decreased lung tumor growth and volume by reduction of VEGFA in human A549 lung tumor xenografts^51^. These studies suggest that tumor-produced VEGF has additional biological functions, which promote tumor cell proliferation and viability.

The protein tyrosine kinases (PTKs) also play a critical role in NF-κB signaling pathways. Our previous studies have shown that Ang1–7 decreases PTK activity in DU-145 cells. Interestingly, while 17-β-estradiol partially reverses this effect, testosterone both disrupts the action of Ang1–7 and leads to an increase in PTK activity. Moreover, the application of PD19234, a specific AT2 receptor antagonist, resulted in the reversal of the changes triggered by testosterone^23^.

Many diseases, including cancer, are known to deregulate the activity of hormones and their receptors. One of the most common cancers in men, prostate cancer, is classified as a hormone-sensitive tumor; i.e. it is dependent on steroid hormones such as androgens and estrogens for growth and survival. The influence of androgens is mediated by AR which is located on the X chromosome at Xq11–12. Estrogens interact with two different forms of the estrogen receptors ERα and ERβ, each encoded by a separate gene localized on different chromosomes: *ESR1* on 6q25.1-q25.2 and *ESR2* on 14q23.2-q23.3, respectively. It has been found previously that *ESR2* is highly expressed in prostate cancer lines, while *ESR1* is expressed in less extend^52,53^. In the present study, the highest level of *AR* expression was observed in LNCaP cells, with a barely detectable level of *AR* mRNA in DU-145 and PC3 cells (data not shown). Previously, Alimirah *et al*.^54^ note that *AR* mRNA levels were low but detectable in PC3, and the levels in DU-145 cells were about 50% lower than those in LNCaP cells^54^.

Our findings indicate that Ang1–7 reduced the expression of *ESR1* and increased that of *ESR2* in prostate cancer cells. As an oncogenic factor, ESR1 promotes cell viability and division and plays a role in PCa inflammation; in contrast, ESR2 plays a protective role in PCa development because of its anti-cancer and pro-apoptotic properties^52,53,55^. The effect of Ang1–7 on androgen receptor mRNA was dependent on the hormonal status of the cell lines: it up-regulated the levels of *AR* mRNA in the androgen-sensitive LNCaP cells but down-regulated in the androgen-irresponsive DU-145 and PC3 cell lines. Interestingly, a similar effect has been previously obtained with AngII^14^.

The role of androgens in EMT regulation are contradictory. While some investigators have reported EMT activation in response to androgen deprivation^56^, others have implicated AR as an stimulator of EMT activation in PCa cells^57,58^. Similarly to the present study, Jacob *et al*.^59^ suggest that AR acts as a positive regulator of *ZEB2* expression in androgen-dependent cells and as a negative regulator in androgen-independent PCa cells. Moreover, the increase in *ZEB2* level was proportional to the decrease in *AR* levels in androgen-independent cells^59^. However, our findings also indicate that PC3 and DU145, known to possess poor *AR* levels, showed higher expression of *ZEB2* than LNCaP cells (data not show). It is seems that this fact might be related to the invasiveness of prostate cancer cells. Furthermore, PC3 and DU-145 cells were found to demonstrate higher expression of the mesenchymal marker vimentin (*VIM*), and lower expression of the epithelial marker E-cadherin (*CDH1*) than LNCaP cells (data not show). Interestingly, downregulation of *CDH1* expression was only observed in DU-145 after Ang1–7 treatment. In the case of LNCaP and PC3 cells, the level of *CDH1* was significantly stimulated. These results are compatible with data from invasiveness assays. Fan *et al*.^60^ suggest that E-cadherin, a key cell-to-cell adhesion molecule associated with the invasion and metastasis of tumor cells, plays an important role in prostate cancer metastasis by influencing the expression of metastasis-associated gene 1 (*MTA1*)^60^. The migration of cancer cells is critically regulated not only by the physical adhesion of cells to each other, but also to the extracellular matrix^61^. Ang1–7 simulation resulted in the adhesion of LNCaP and DU-145 to fibronectin, a major component of the stromal extracellular matrix, but not PC3 cells, which adhesion to all tested extracellular matrix proteins decreased. In a study of the effect of RGD peptide on prostate cancer cell adhesion, Sakko *et al*.^62^ noted that DU-145 was more sensitive in its reaction to fibronectin that PC3^62^.

Other researchers have also reported modulation of cell adhesion to the ECM by angiotensins. We observed previously that AngII improved ability of prostate cancer cells to adhere to the ECM^13^. Many other studies have demonstrated that Ang1–7 has an antagonistic effects than Ang II. The cancer cells spread to new organs via the lymph or blood stream, and hence require the ability to survive and grow without contact with the ECM or neighboring cells. Our results clearly show that Ang1–7 can inhibit the anchorage-independent growth and non-adherent colony formation of both LNCaP and DU-145 cells, but not PC3. In contrast, Ang II was earlier observed to have a statistically insignificant stimulatory effect in all tested prostate cancer cells^13^.

In summary, the results confirm the existence of complicated dependence networks between the various elements of the local RAS and the molecular and cellular mechanisms of prostate cancerogenesis. Ang1–7 can decrease the level of cancer malignancy; however, its effects depend on the dose and time of incubation, as well as on the aggressiveness and hormonal status of cells. The influence of Ang1–7 is mediated not only by MAS angiotensin receptors but also AT2, and its pleiotropic action is associated with the modulation of many different signaling pathways, such as NF-κB, or with the regulation of the multiple genes associated with sex steroid receptors.

## Electronic supplementary material

SUPPLEMENTARY MATERIAL

## Data Availability

Authors declare that all data generated or analyzed during this study are included in this published article. If any interest needs more details please contact corresponding author by private email.
