# Supplementary material for: Angiotensin 1–7 modulates molecular and cellular processes central to the pathogenesis of prostate cancer
Source: Sci Rep. 2018 Oct 25;8:15772. doi: 10.1038/s41598-018-34049-8 (PMC6202343; doi:10.1038/s41598-018-34049-8)

SUPPLEMENTARY MATERIAL

TITLE:
ANGIOTENSIN 1-7 MODULATES MOLECULAR AND CELLULAR PROCESSES CENTRAL TO THE PATHOGENESIS OF PROSTATE CANCER.

Kamila Domińska[1*], Piotr Okła[1], Karolina Kowalska[2], Dominika Ewa Habrowska-Górczyńska[2], Kinga Anna Urbanek[1,2], Tomasz Ochedalski[1] and Agnieszka Wanda Piastowska-Ciesielska[1,2]

Department of [1]Comparative Endocrinology and [2]Laboratory of Cell Cultures and Genomic Analysis, Medical University of Lodz, Lodz 90-752, Poland

* Coresponding author: Kamila Domińska PhD (email:kamila.dominska@umed.lodz.pl)

WOUND HEALING ASSAY

LNCAP

I.

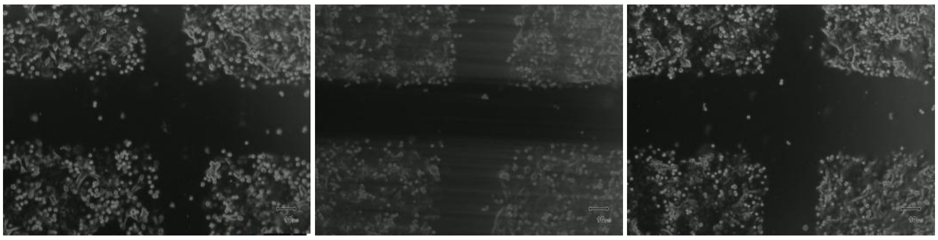

Control/0h

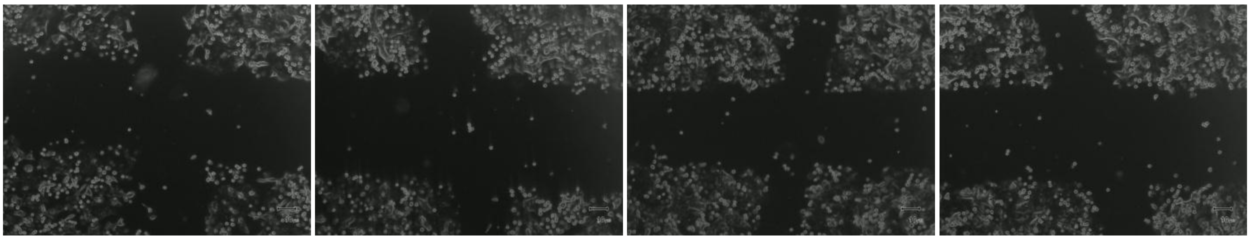

Ang1-7/0h

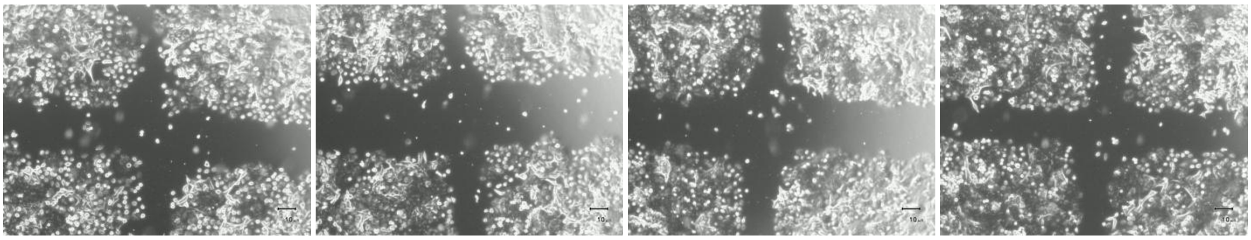

Control/24h

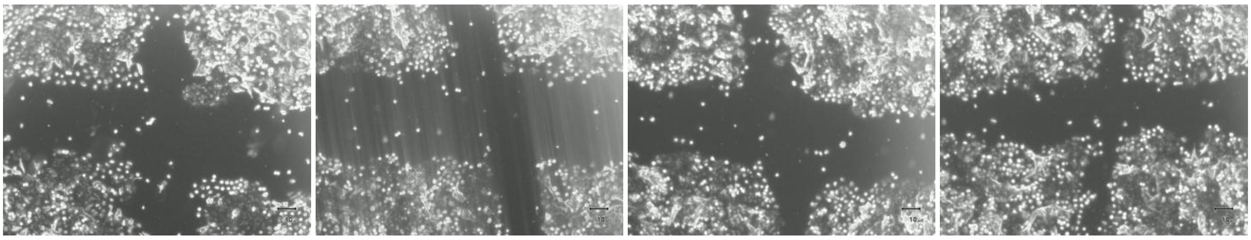

Ang1-7/24h

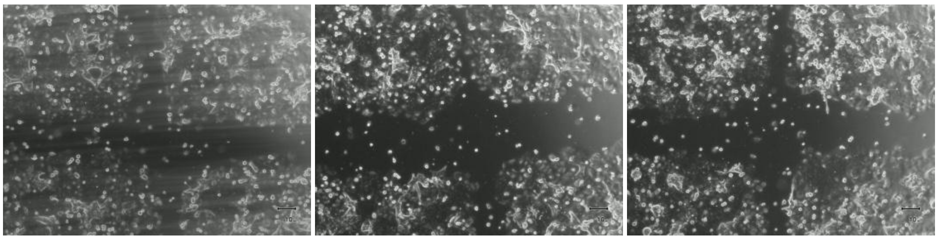

Control/48h

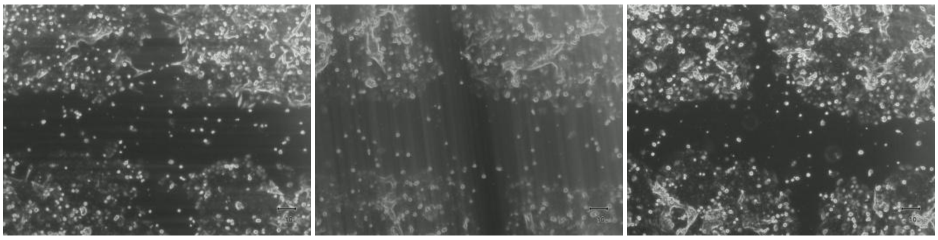

Ang1-7/48h

LNCAP

II.

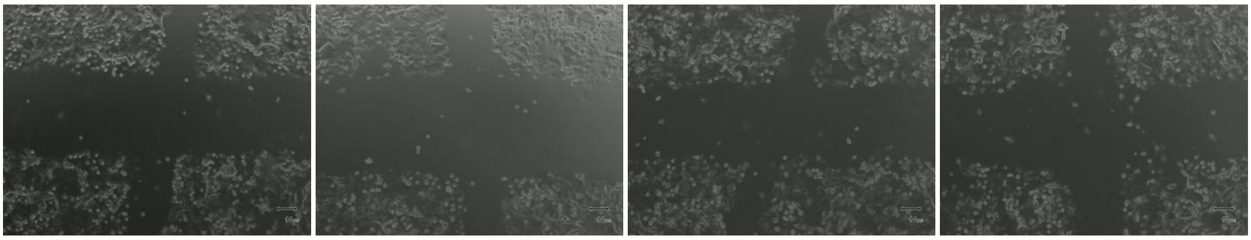

Control/0h

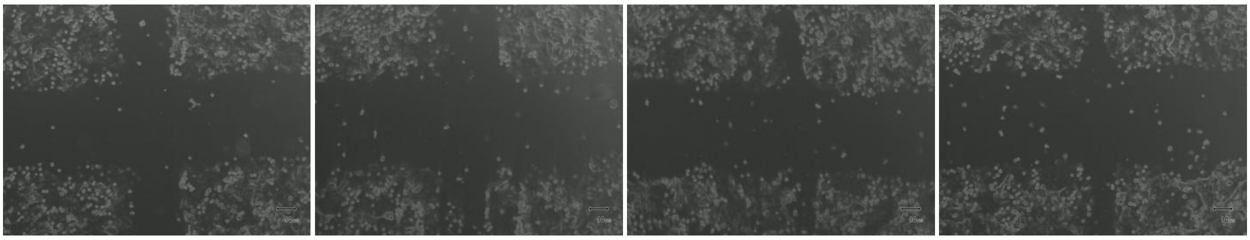

Ang1-7/0h

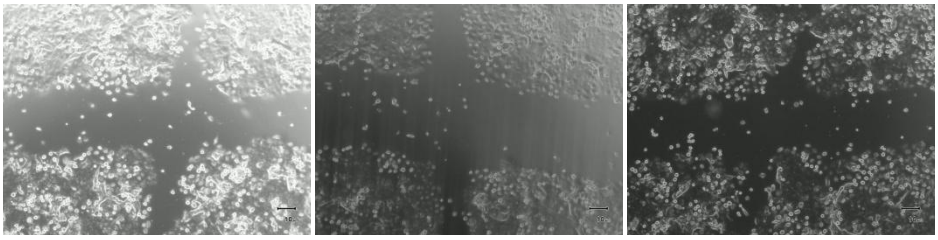

Control/24h

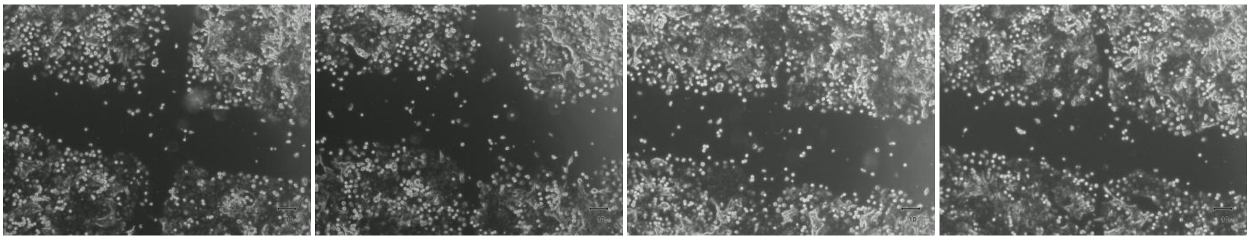

Ang1-7/24h

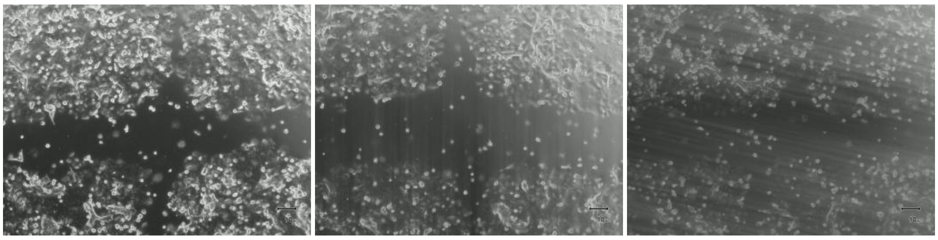

Control/48h

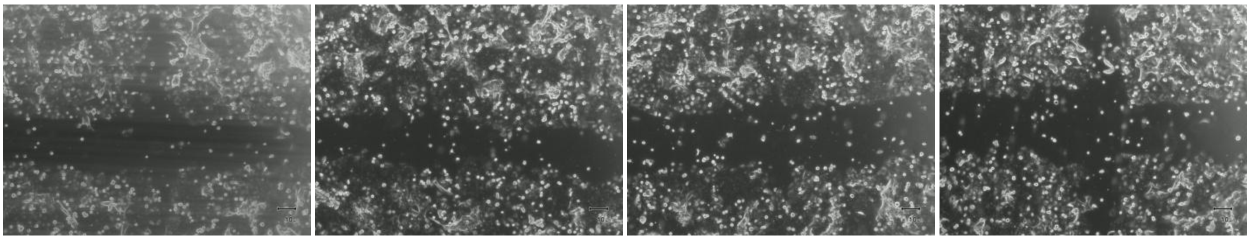

Ang1-7/48h

DU-145

I.

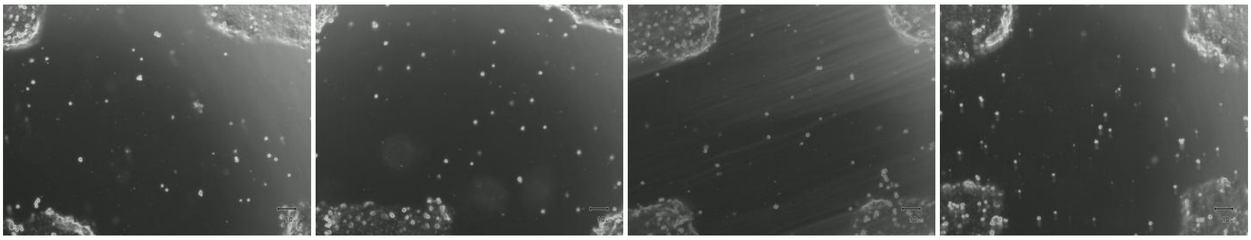

Control/0h

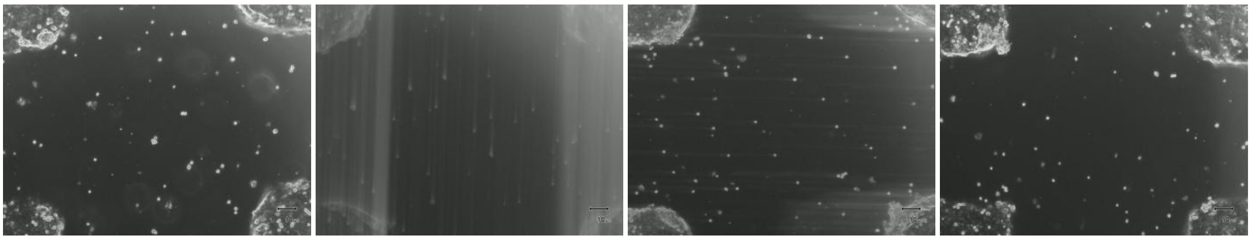

Ang1-7/0h

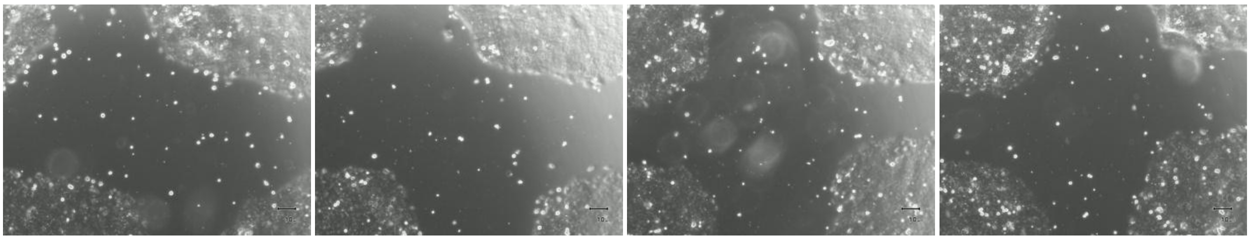

Control/24h

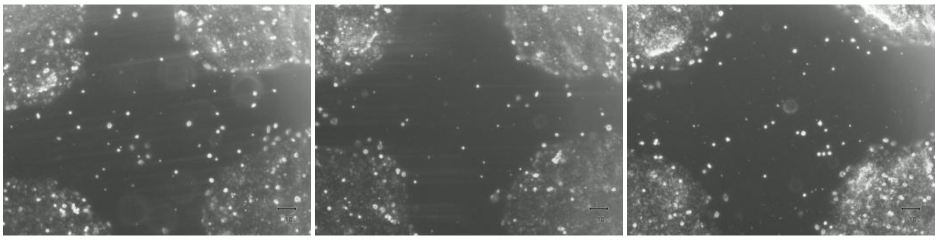

Ang1-7/24h

SUPPLEMENTARY MATERIAL

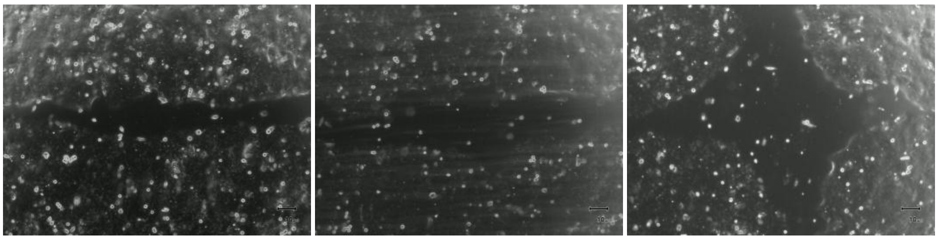

Control/48h

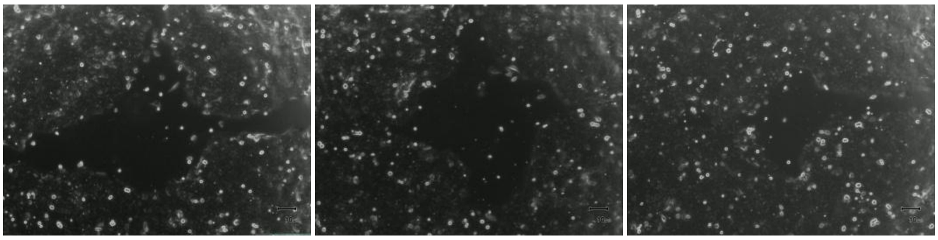

Ang1-7/48h

DU-145

II.

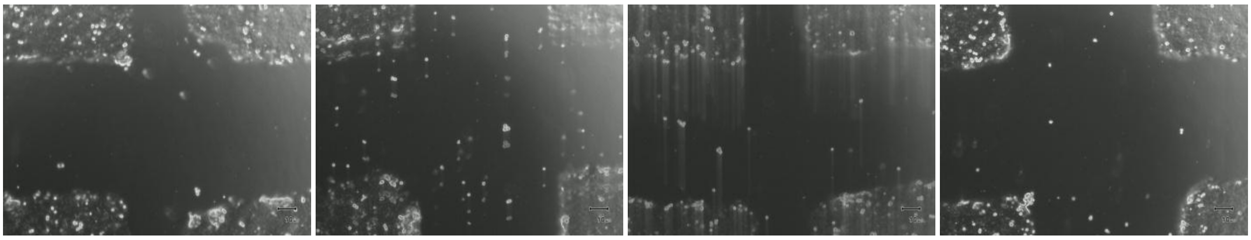

Control/0h

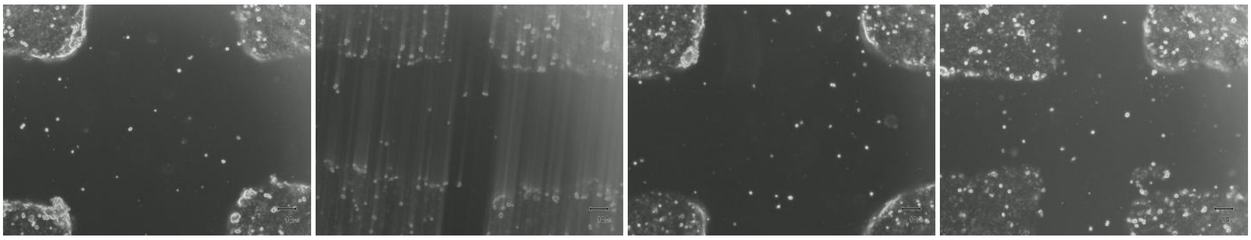

Ang1-7/0h

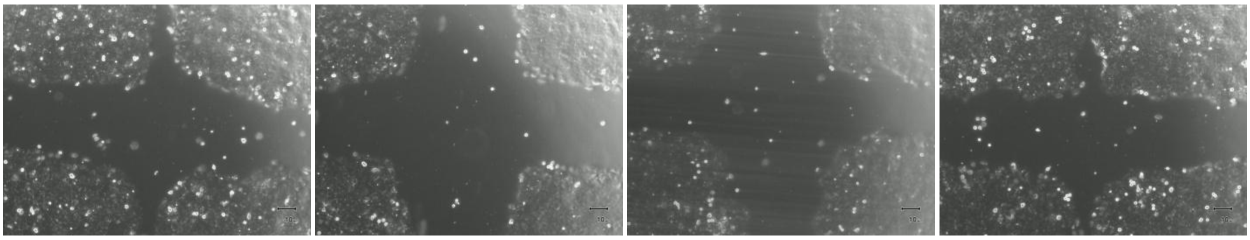

Control/24h

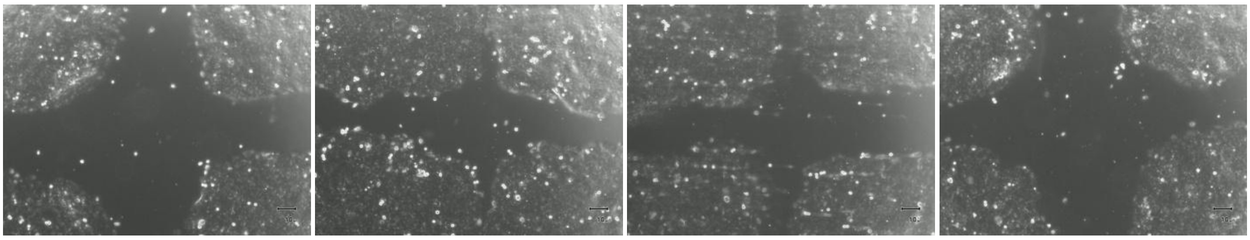

Ang1-7/24h

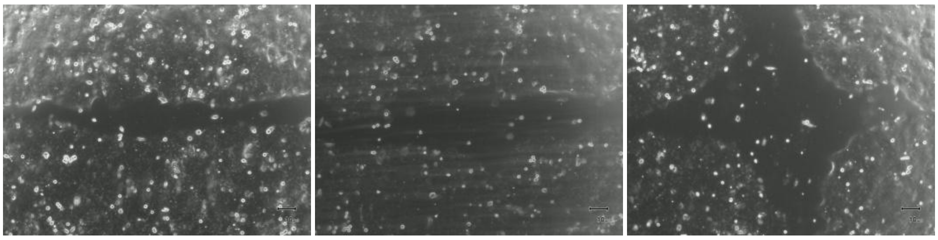
Control/48h

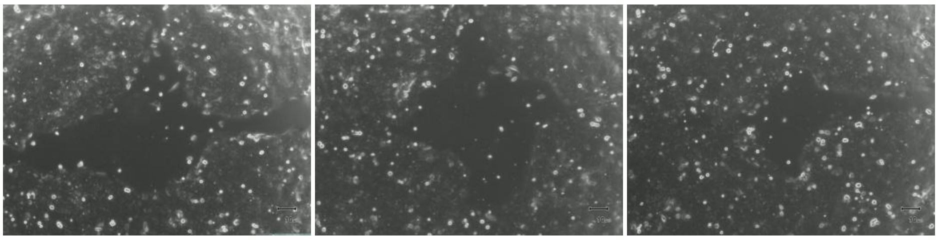
Ang1-7/48h

PC3

I.

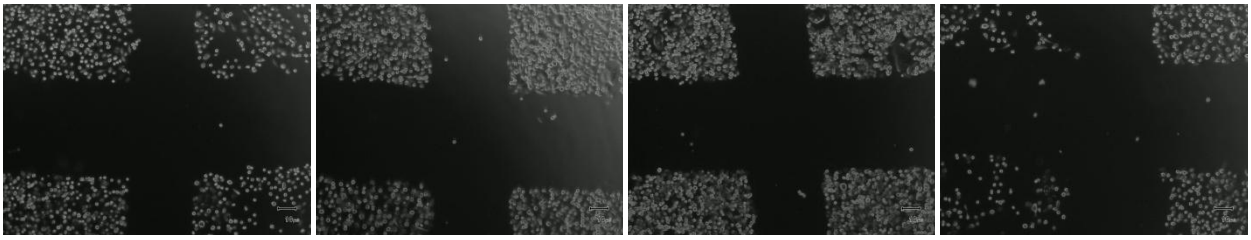
Control/0h

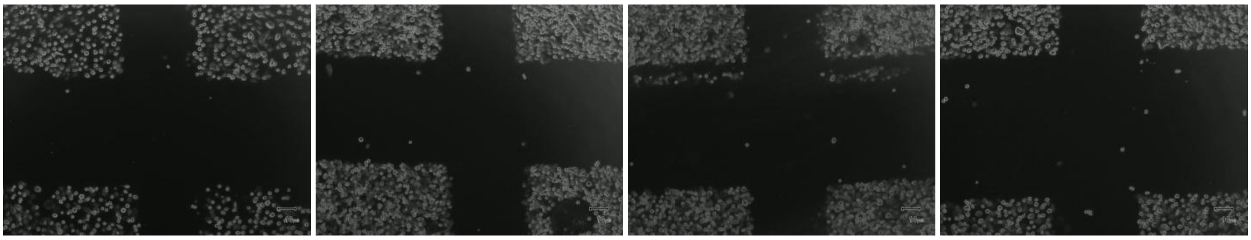
Ang1-7/0h

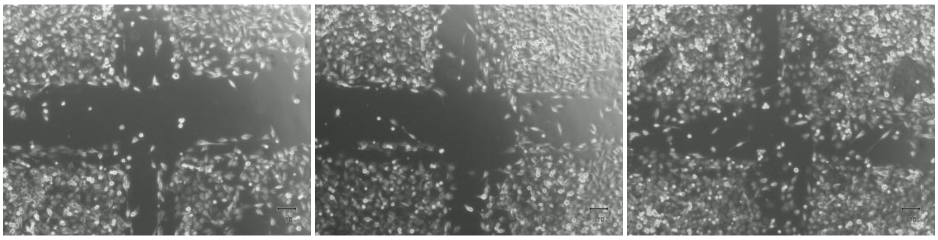
Control/24h

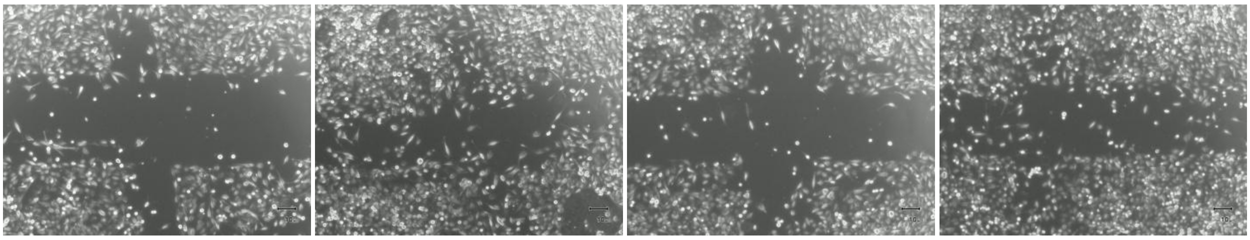
Ang1-7/24h

SUPPLEMENTARY MATERIAL

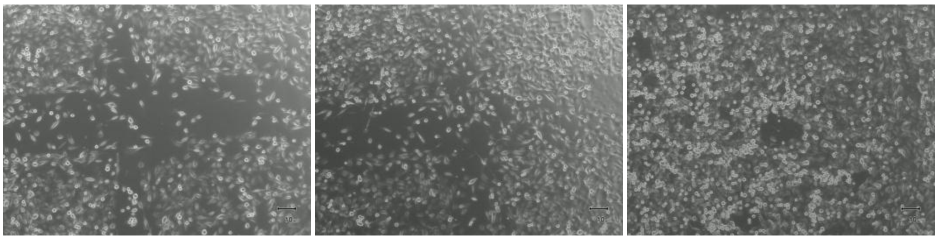

Control/48h 1x100%

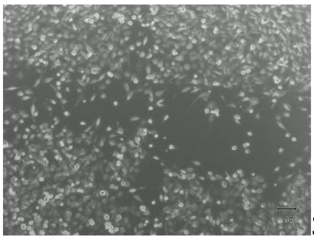

Ang1-7/48h 3x100%

PC3

II.

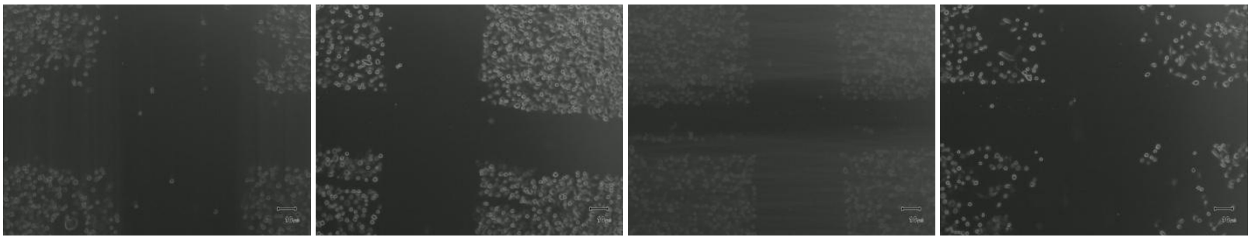

Control/0h

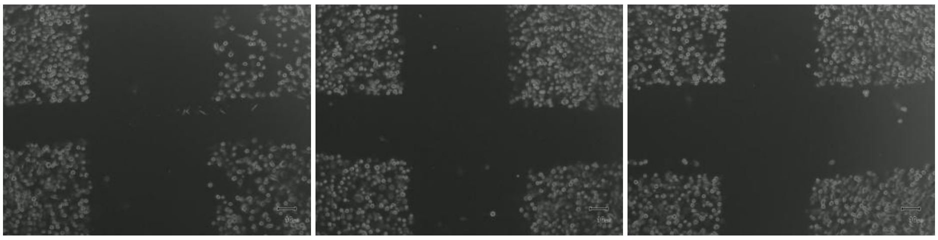

Ang1-7/0h

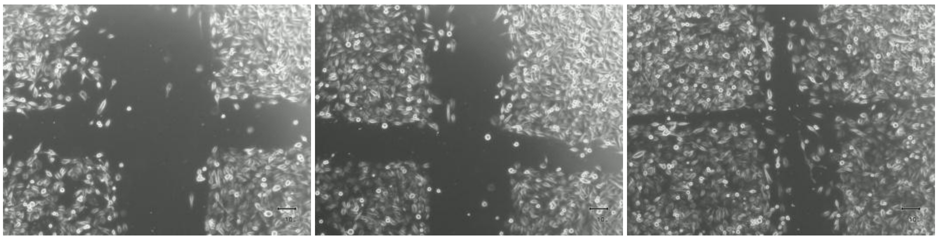

Control/24h

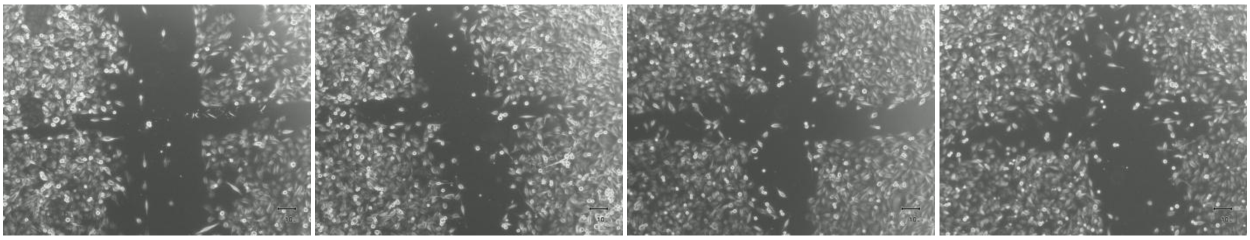

Ang1-7/24h

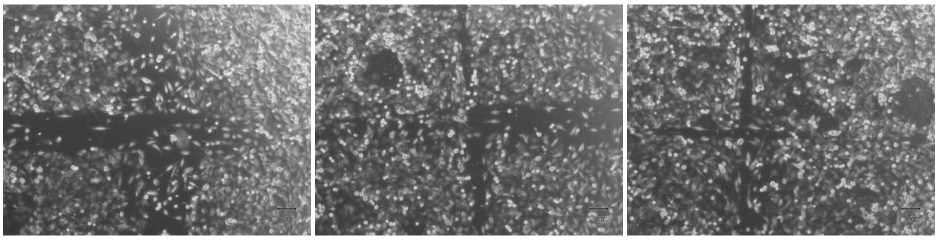

Control/48h 1x100%

Ang1-7/48h 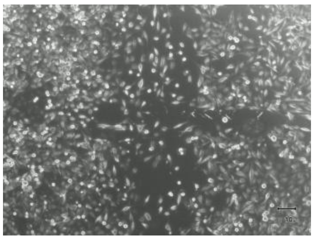 3x100%

PC3

III.

Control/0h 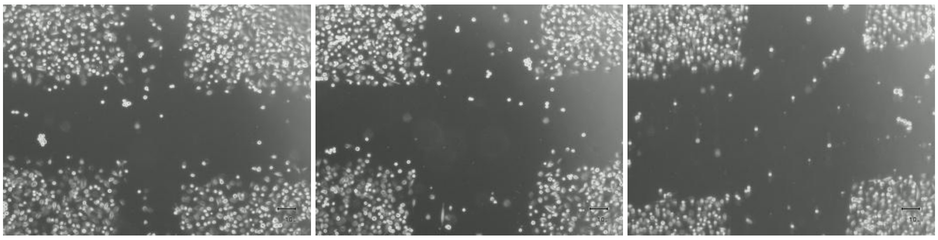

Ang1-7/0h 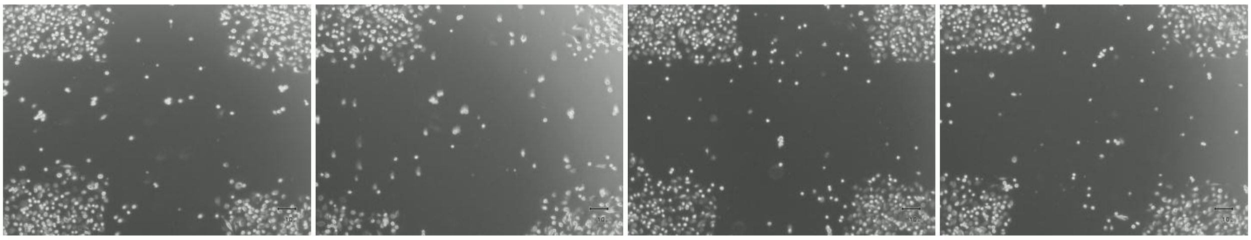

Control/24h 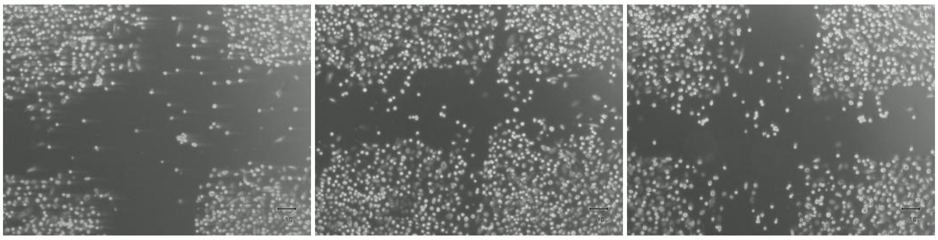

Ang1-7/24h 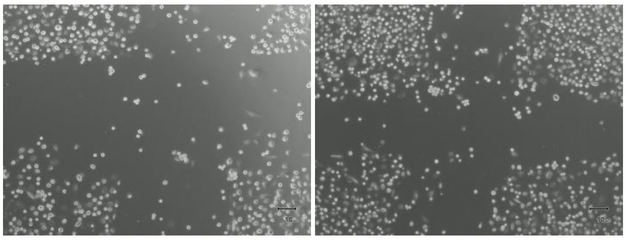

Control/48h 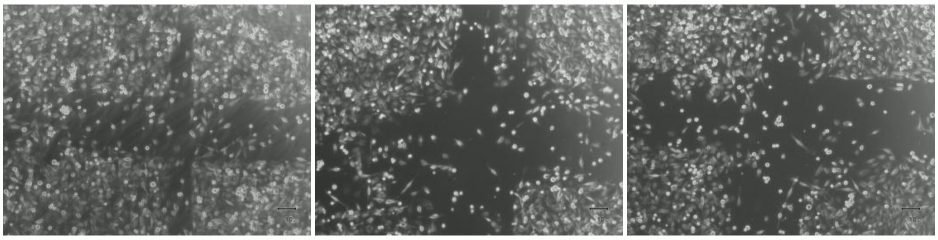 1x100%

Ang1-7/48h 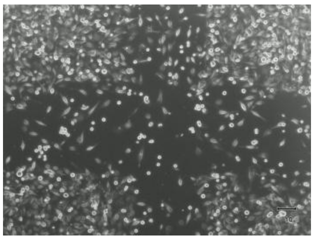 3x100%

SUPPLEMENTARY MATERIAL

PC3

IV.

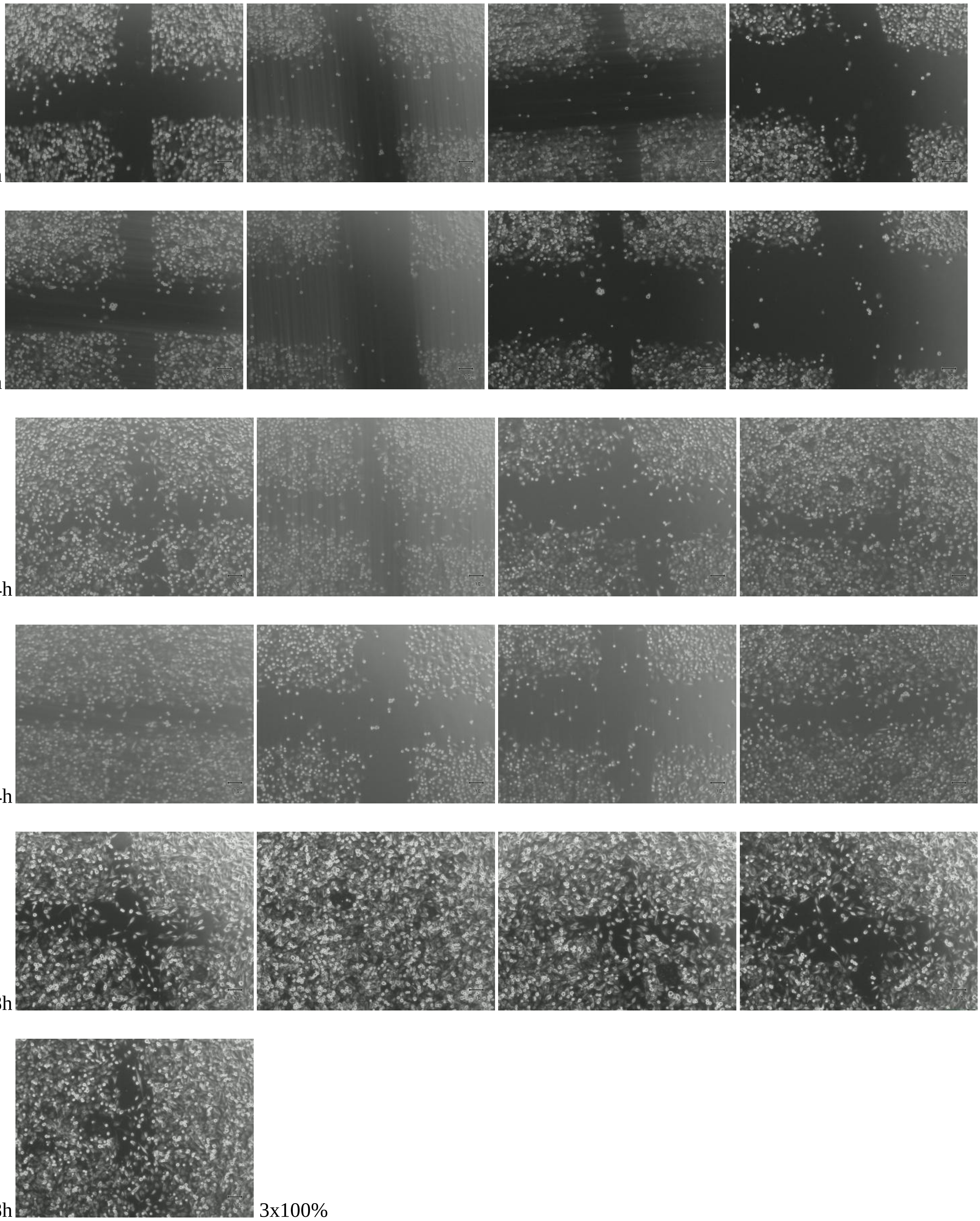

Control/0h

Ang1-7/0h

Control/24h

Ang1-7/24h

Control/48h

Ang1-7/48h

3x100%

SUPPLEMENTARY MATERIAL

GELATIN ZYMOGRAPHY ASSAY

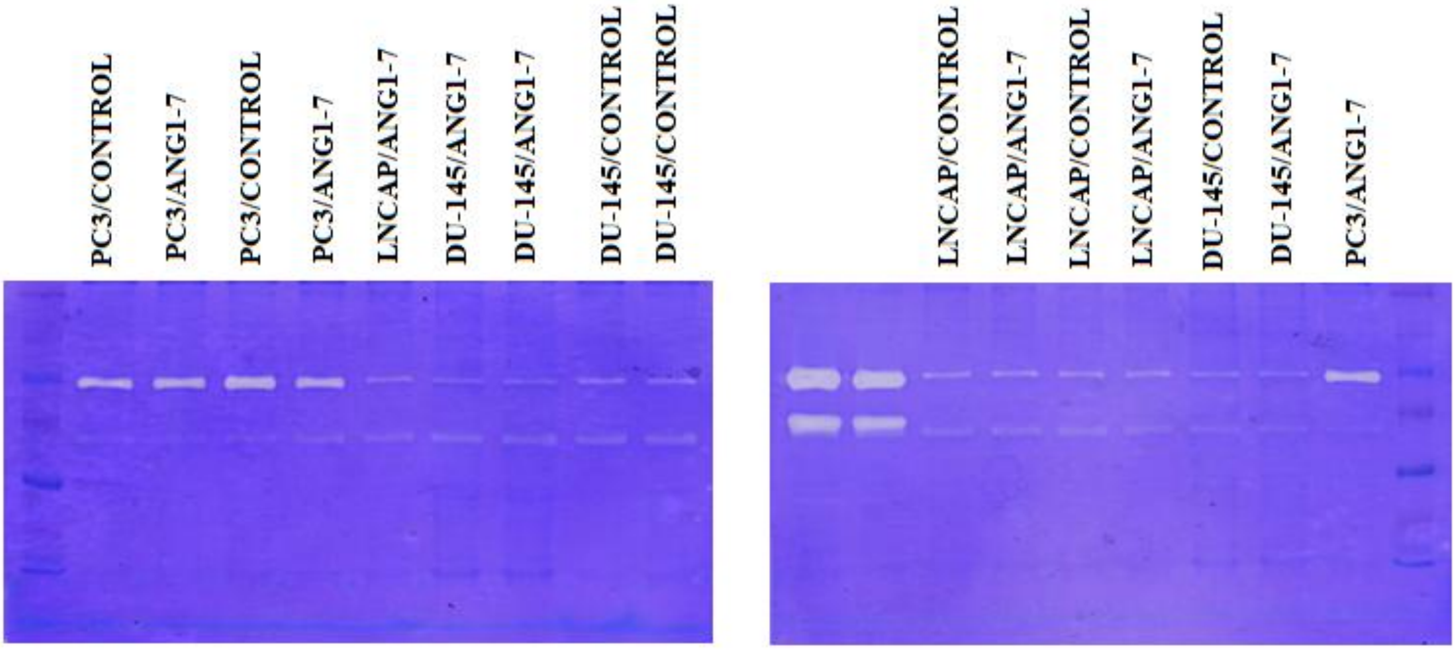

SOFT AGAR COLONY FORMATION ASSAY

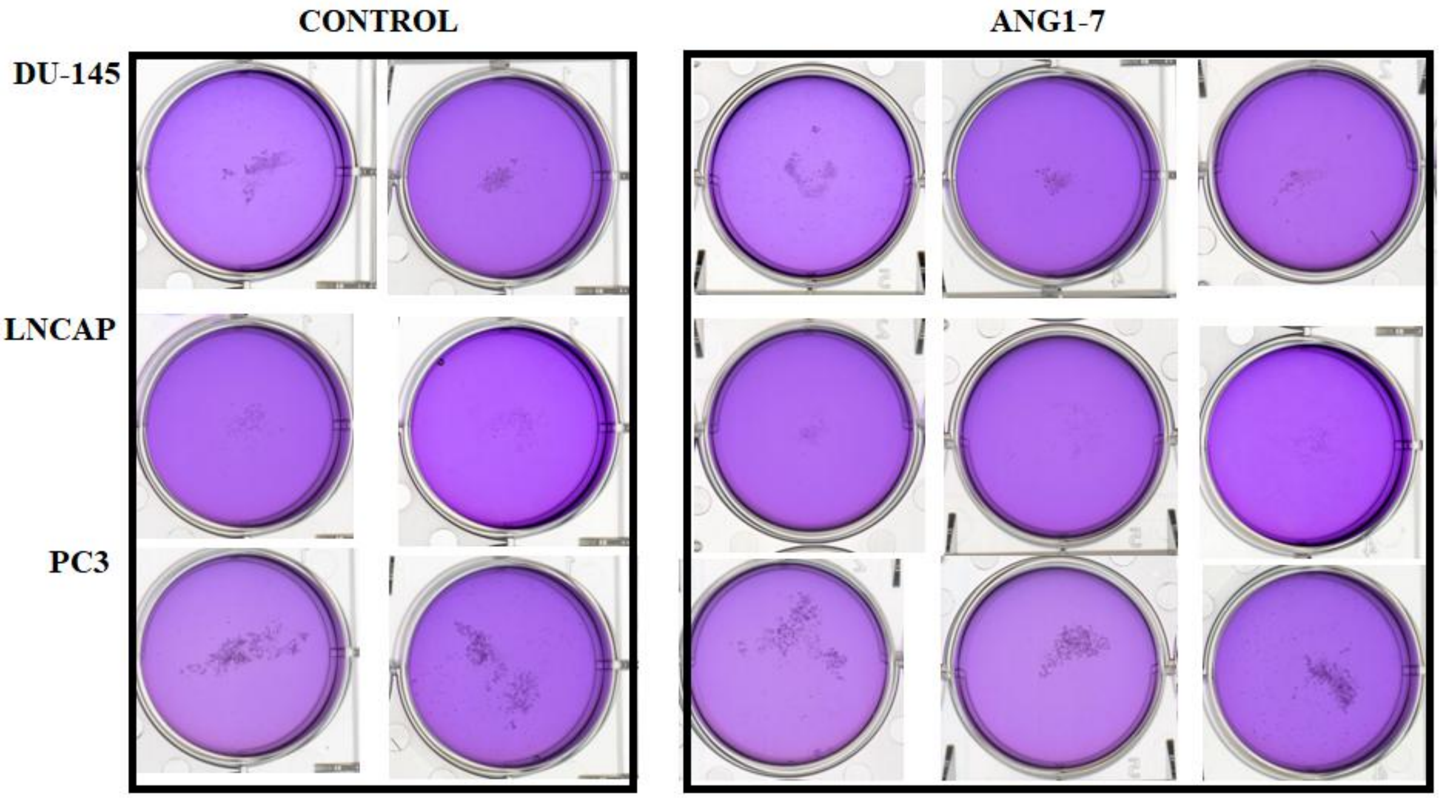

Supplement: Supplementary file 1 — SUPPLEMENTARY MATERIAL [file 41598_2018_34049_MOESM1_ESM.pdf]
